# l-2-Hydroxyglutarate impairs neuronal differentiation through epigenetic activation of *MYC* expression

**DOI:** 10.1172/JCI197010

**Published:** 2026-03-17

**Authors:** Wen Gu, Xun Wang, Ashley Solmonson, Ling Cai, Yi Xiao, Alpaslan Tasdogan, Jordan Franklin, Yuannyu Zhang, Hua Zhang, Aundrea K. Westfall, Ashley Rowe, Hetali Trivedi, Brandon Faubert, Zheng Wu, Jessica Sudderth, Lauren G. Zacharias, Bushra Afroze, Ilya Bezprozvanny, Sunil Sudarshan, Feng Cai, Samuel K. McBrayer, Thomas P. Mathews, Ralph J. DeBerardinis

**Affiliations:** 1 Children’s Medical Center Research Institute,; 2 Quantitative Biomedical Research Center, Peter O’Donnell Jr. School of Public Health,; 3 Department of Physiology, and; 4 Department of Pathology, University of Texas Southwestern Medical Center, Dallas, Texas, USA.; 5 Department of Paediatrics & Child Health, The Aga Khan University, Karachi, Pakistan.; 6 Department of Urology, University of Alabama at Birmingham, Birmingham, Alabama, USA.; 7 Peter O’Donnell Jr. Brain Institute,; 8 Eugene McDermott Center for Human Growth and Development, and; 9 Howard Hughes Medical Institute, University of Texas Southwestern Medical Center, Dallas, Texas, USA.

**Keywords:** Clinical Research, Development, Metabolism, Epigenetics, Human stem cells, Neurodevelopment

## Abstract

High levels of l- and d-2-hydroxyglutarate (2HG), the reduced forms of α-ketoglutarate (αKG), are implicated in neurodevelopmental disorders and cancer by modulating αKG-dependent dioxygenases involved in histone, DNA, and RNA demethylation. L-2HG dehydrogenase (L2HGDH) deficiency, a rare autosomal recessive inborn error of metabolism associated with systemic L-2HG elevation, causes progressive neurological disability and increased brain tumor risk of unclear mechanism. Using an isogenic, patient-derived induced pluripotent stem cell system, we examined the impact of L2HGDH deficiency on neural progenitor cell (NPC) function and neuronal differentiation. L2HGDH deficiency caused L-2HG accumulation, NPC hyperproliferation, increased clonogenicity, and defective neuronal differentiation in 2D cultures and cortical spheroids. Editing the *L2HGDH* locus to WT reversed these effects. Inhibiting glutaminase reduced L-2HG levels and induced neuronal differentiation. L-2HG–dependent inhibition of KDM5 histone demethylases led to widespread retention of H3K4me2/3, markers of active gene expression, with prominent enrichment at the *MYC* locus and elevated *MYC* expression across multiple neural cell types. Despite broadly altered histone methylation, genetically or pharmacologically normalizing *MYC* completely restored neuronal differentiation. These data indicated that a primary metabolic disturbance activated *MYC* to favor self-renewal and suppress neuronal lineage commitment.

## Abstract 2

Patient-derived stem cells and cerebral organoids reveal that L2HG epigenetically activates MYC, impairing neuronal differentiation—reversible by gene editing, L2HG depletion or MYC inhibition.

## Introduction

l-2-Hydroxyglutaric aciduria (L-2HGA) is a rare autosomal recessive inborn error of metabolism (IEM) caused by mutations in *L2HGDH* (1), the gene encoding l-2-hydroxyglutarate dehydrogenase, an oxidoreductase that interconverts L-2HG and α-ketoglutarate (αKG) (2). Individuals with L-2HGA present with developmental delay, seizures, and progressive neurological decline (3), along with an elevated risk of tumors in the CNS (4) and elsewhere (5). These neurodevelopmental abnormalities and malignancies imply altered cellular differentiation and proliferation. Despite the identification of L-2HG as a hallmark metabolite in these patients, the underlying mechanisms connecting its accumulation to impaired neuronal function are poorly understood. Unlike many other IEMs associated with neurodevelopmental disabilities, patients with L-2HGA do not display acute encephalopathic crises or overt signs of systemic metabolic dysfunction, such as acidosis, hypoglycemia, or hyperammonemia.

2-Hydroxyglutarate (2HG) exists in 2 enantiomeric forms, L-2HG and D-2HG, each with distinct biological and pathological effects. Most mechanistic evidence connecting 2HG to disease stems from studies on D-2HG in cancer, particularly in gliomas (6) and acute myeloid leukemia (7). D-2HG accumulates as a result of mutations in isocitrate dehydrogenase-1 (IDH1) or IDH2 (8, 9), which confer a neomorphic enzymatic activity that converts αKG to D-2HG. This accumulation inhibits αKG-dependent dioxygenases (10), including histone demethylases (11) and the Ten-eleven translocation (TET) family of hydroxylases that promote DNA demethylation (12). This enzymatic inhibition drives hypermethylation, promoting tumorigenesis and blocking differentiation. These findings have established D-2HG as an oncometabolite and a central player in epigenetic reprogramming associated with malignancy.

Elevated L-2HG levels have been observed in a variety of contexts, including in clear-cell renal cell carcinoma due to reduced L2HGDH activity (13, 14) as well as in metabolic stress scenarios such as hypoxia (15–17), acidosis (18), and electron transport chain dysfunction (19). L-2HG accumulation is also observed in other IEMs, such as lipoyltransferase-1 deficiency (20). This metabolite is increasingly recognized for its effects on development and regenerative processes outside of cancer. In mouse blastocysts, elevated L-2HG levels inhibit histone demethylases, resulting in widespread histone methylation and transcriptional dysregulation after fertilization (21). During larval growth in *Drosophila*, timed accumulation and degradation of L-2HG are necessary for proper epigenetic regulation of gene expression, suggesting a developmental role for this metabolite (22). The effects of L-2HG appear highly context dependent. For example, glial-derived L-2HG promotes axon regeneration by acting on neuronal metabotropic GABA_B_ receptors to enhance cAMP signaling. Conversely, increased L-2HG within neurons negatively regulates neuroregeneration in the *Drosophila* CNS (23).

Despite these advances, the mechanisms by which L-2HG interferes with neuronal function in humans remain poorly understood. Here, we investigate the impact of L-2HG on neuronal differentiation using patient-derived, isogenic neural progenitor cells (NPCs) with either functional or deficient L2HGDH. We conduct metabolic, transcriptional, and epigenetic characterization, alongside functional perturbations and analyses in both 2D and organoid neuronal differentiation models. In so doing, we reveal the metabolic and functional consequences of L-2HG excess during neuronal specification, identify the molecular mechanism driving these abnormalities, and demonstrate improved neuronal differentiation and function upon reversal of L-2HG’s effects on key epigenetic targets.

## Results

### Genomic and metabolic analysis of patients with L-2HGA.

**Figure 1:**
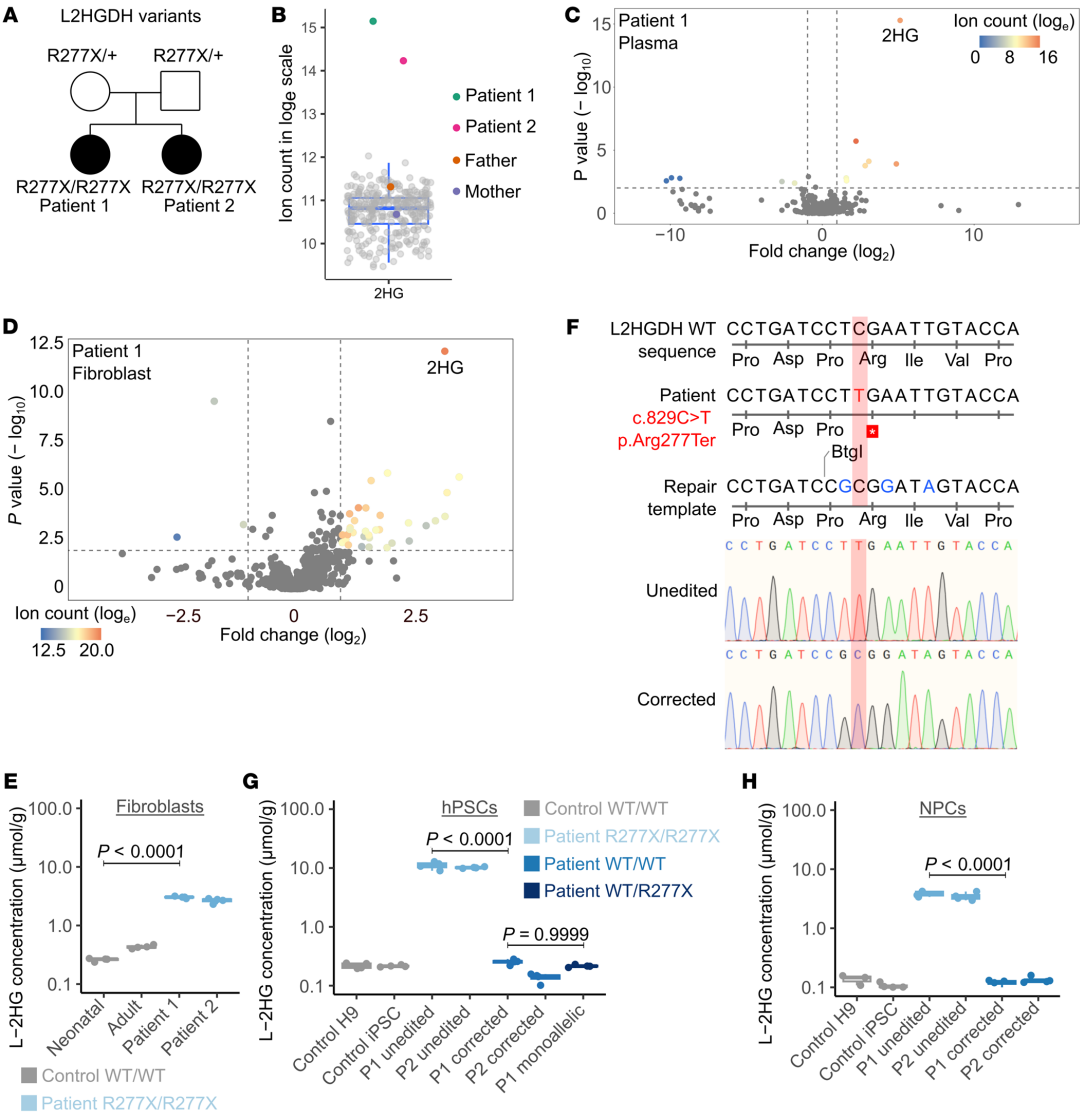
An isogenic, patient-derived iPSC system to study L2HGDH deficiency. (**A**) *L2HGDH* genotypes of 2 L-2HGA patients and their parents. (**B**) Relative 2HG abundance in plasma from L-2HGA patients, parents, and unrelated subjects. (**C**) Volcano plot of plasma metabolites comparing patient 1 to unrelated subjects (*n* = 427; sibling excluded). A linear mixed-effects model was used to account for repeated measures per individual, with subject ID as a random effect. *P* values were adjusted for multiple testing (Benjamini-Hochberg), with statistical significance thresholds of adjusted *P* < 0.01 and fold change > 2 or < 0.5. (**D**) Volcano plot of fibroblast metabolites comparing patient 1 to 29 unrelated lines (sibling excluded), each profiled in quadruplicate. A mixed-effects model was used as in **C**, with cell line as the random effect. (**E**) L-2HG concentrations in neonatal control, adult control, and patient fibroblasts. (**F**) Alignment showing the pathogenic *L2HGDH* variant (c.829C>T, red) and edits (blue) introduced by the ssODN repair template. Electropherograms show unedited and biallelically corrected iPSC alleles. (**G** and **H**) L-2HG levels in PSCs (**G**) and NPCs (**H**) from control and patient lines with unedited or corrected *L2HGDH* alleles. In **G**, “hPSC” denotes human pluripotent stem cells and is used to collectively refer to the H9 human embryonic stem cell line and the patient-derived human iPSC lines. For **B**, **E**, **G**, and **H**, data are shown as box-and-whisker plots with jittered points (*n* = 4 biological replicates for **E**, **G**, and **H**); boxes represent the 25th–75th percentile, horizontal lines indicate medians, and whiskers extend to 1.5× the interquartile range. Significance was determined by 1-way ANOVA with Tukey’s HSD test.

We identified a homozygous loss-of-function variant in *L2HGDH* (c.829C>T; p.Arg277Ter) in 2 siblings diagnosed with L-2HGA in Pakistan (Figure 1A, patients 1 and 2). We confirmed that both siblings inherited 1 pathogenic variant from each heterozygous carrier parent, consistent with the known autosomal recessive inheritance of this disease (2). Metabolomic profiling of plasma revealed elevated 2HG levels in the patients relative to their parents and control subjects (Figure 1B). Metabolite differential analysis demonstrated that 2HG was by far the most statistically significantly altered metabolite in the plasma of the L-2HGA patients (Figure 1C). We also performed metabolomics in fibroblast cultures from both siblings and observed marked 2HG accumulation compared with fibroblasts from 29 controls (Figure 1D and Supplemental Figure 1A; supplemental material available online with this article; https://doi.org/10.1172/JCI197010DS1). We then quantified the levels of both 2HG enantiomers in fibroblasts from the patients and controls; this confirmed elevated L-2HG in the patients’ cells, with no accumulation of D-2HG, as expected (Figure 1E and Supplemental Figure 1B).

### CRISPR/Cas9-mediated correction of L2HGDH mutation.

To model L-2HGA in culture and explore the molecular basis of neuronal dysfunction, we generated induced pluripotent stem cells (iPSCs; Supplemental Figure 1C) from patient fibroblasts using the reprogramming factors OCT4, KLF4, SOX2, and c-MYC introduced via a nonreplicating Sendai virus (24). Eight clonal cell lines were derived from the reprogrammed fibroblasts for each donor, and 2 of these lines from each donor were further expanded and characterized. We then applied CRISPR/Cas9-mediated genome editing to correct the c.829C>T mutation in patient-derived iPSCs using a single-stranded oligodeoxynucleotide (ssODN) repair template (Figure 1F and Supplemental Figure 1D). Sanger sequencing confirmed correction of *L2HGDH* in patient-derived iPSC clones, demonstrating precise incorporation of blocking mutations (in blue in Figure 1F) to prevent Cas9 recutting (25). In subsequent experiments, “corrected” clones are ones in which *L2HGDH* was edited to the WT sequence, and “unedited” clones are ones that were subjected to the editing workflow but did not become edited (Supplemental Figure 1D). We measured L-2HG concentrations in pluripotent stem cells from control subjects and from corrected and unedited iPSCs from both patients (Figure 1G). Unedited iPSCs retained high L-2HG levels, but edited iPSCs had L-2HG levels comparable to control hPSCs. Consistent with autosomal recessive inheritance of L-2HGA, monoallelic homology-directed recombination reduced intracellular L-2HG to levels comparable to biallelically corrected iPSCs (Figure 1G). We then differentiated these iPSCs into NPCs (Supplemental Figure 1C) according to a well-established dual-SMAD inhibition neural specification protocol (26) and found that unedited NPCs from both patients had high L-2HG levels but corrected NPCs had low L-2HG levels comparable to NPCs from controls (Figure 1H). D-2HG levels were comparable in all these cell lines (Supplemental Figure 1E).

### L2HGDH-mutant NPCs are hyperproliferative with enhanced self-renewal capacity and impaired differentiation to neurons.

**Figure 2:**
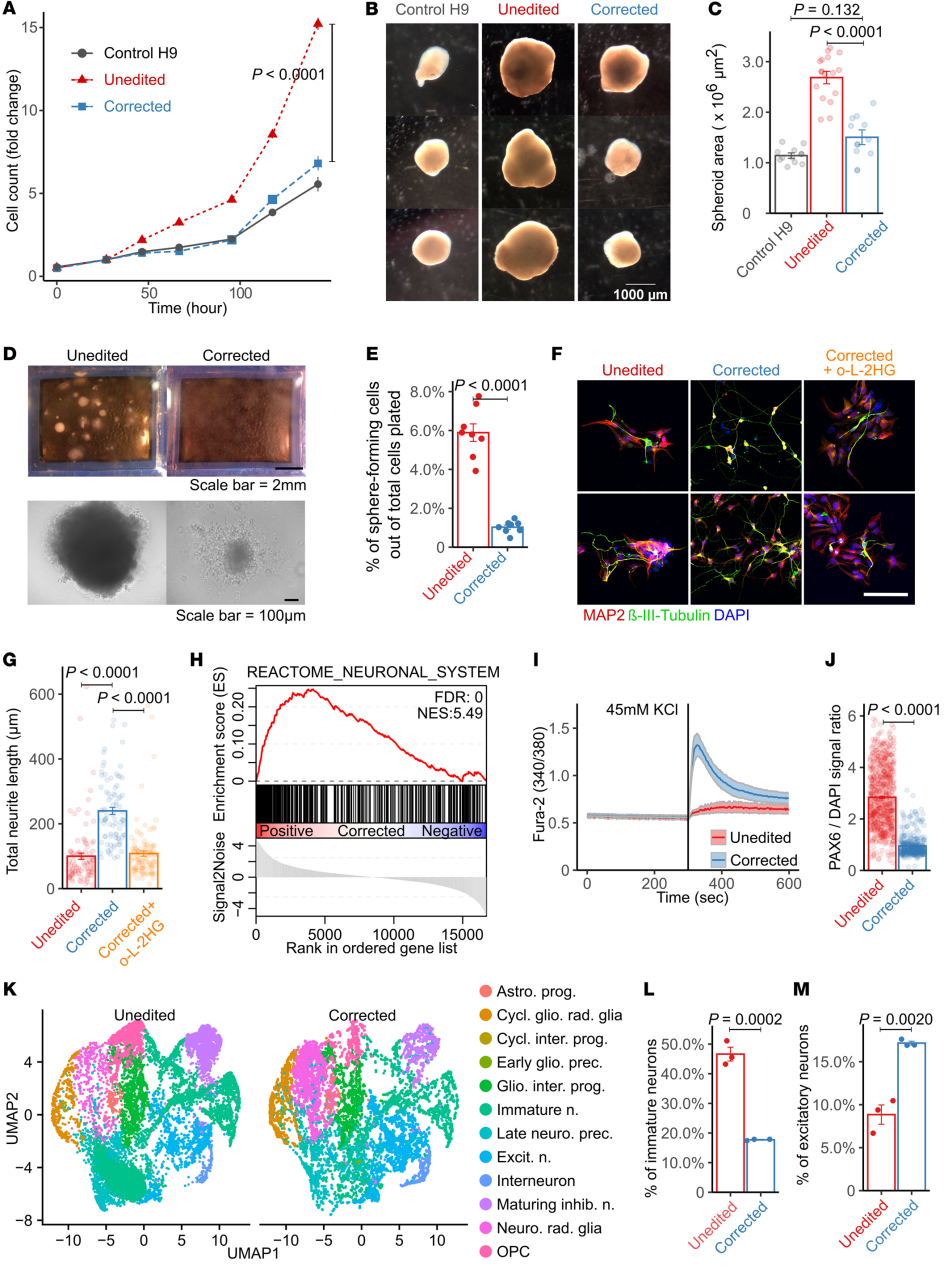
L-2HG accumulation enhances NPC proliferation and self-renewal while inhibiting neuronal differentiation. (**A**) Growth curves of NPCs derived from H9 control, unedited, and corrected iPSCs. (**B**) Stereoscopic images of cortical spheroids at day 30 of differentiation. Scale bar: 1,000 μm. (**C**) Quantification of spheroid surface area at day 30. (**D**) Images of colonies formed from single NPCs. Scale bars: 2 mm (top), 100 μm (bottom). (**E**) Frequency of colony formation in single NPCs. (**F**) Immunostaining for MAP2 and β-III-tubulin in unedited, corrected, and corrected + 30 μM octyl-L-2HG (o-L-2HG) neurons at day 14. DAPI marks nuclei. Scale bar: 100 μm. (**G**) Quantification of neurite length from images. (**H**) GSEA plot showing enrichment of the REACTOME_NEURONAL_SYSTEM gene set in corrected versus unedited neurons. (**I**) Fura-2 ratio recordings of Ca^2+^ dynamics during 45 mM KCl application in neurons differentiated for 47 days (unedited: *n* = 29; corrected: *n* = 24). (**J**) Quantification of PAX6 signal intensity normalized to DAPI in unedited and corrected neurons. (**K**) UMAP of single-cell transcriptomes from day 45 cortical spheroids (unedited and corrected; 12,660 cells each). Annotated populations include progenitors (prog.), precursors (prec.), neurons (n.), astrocyte (astro.) lineage, radial (rad.) glia, intermediate (inter.) progenitors, excitatory (excit.) neurons, inhibitory (inhib.) neurons, interneurons, and oligodendrocyte progenitor cells (OPC). (**L** and **M**) Proportions of immature neurons (**L**) and excitatory neurons (**M**) among annotated populations. For **A**, **C**, **E**, **G**, **J**, **L**, and **M**, data are shown as mean ± SEM. Significance was determined by 2-way ANOVA for **A**, 1-way ANOVA with Tukey’s HSD test for **C** (*n* = 10 H9, *n* = 15 unedited, *n* = 10 corrected) and **G** (*n* = 96 unedited, *n* = 86 corrected, *n* = 108 corrected + o-L-2HG), and unpaired 2-tailed Student’s *t* test for **E** (*n* = 8), **J** (*n* = 679 unedited, *n* = 430 corrected), and **L** and **M** (*n* = 3 each).

Unedited NPCs were highly proliferative, exceeding the proliferation rates of corrected NPCs and control H9 NPCs (Figure 2A). We generated cortical spheroids from H9, unedited, and corrected NPCs because these 3D models of differentiation can be used to study disease processes that impact the brain (27). After 30 days of differentiation, spheroids derived from unedited NPCs were much larger than those from corrected and control NPCs (Figure 2B). Quantification of spheroid surface area confirmed the increased size of unedited relative to corrected spheroids, whereas no difference was detected between corrected and control spheroids (Figure 2C). Next, to evaluate NPC self-renewal capacity, we cultured single NPCs and measured their ability to form colonies (28). Stereoscopic and phase-contrast imaging revealed enhanced colony formation in unedited NPCs compared with corrected cells (Figure 2D). Quantification of sphere-forming frequency demonstrated a large increase in the proportion of sphere-forming cells in unedited NPCs relative to corrected lines (Figure 2E).

Next, we investigated the effects of *L2HGDH* mutation on neuronal differentiation by subjecting patient-derived NPCs to a well-described neuronal differentiation protocol (Supplemental Figure 1C) (29). This approach generates a majority of VGLUT1^+^ neurons, which are presumed to be glutamatergic forebrain neurons (26). We immunostained day 14 2D neuronal cultures with MAP2 (Cell Signaling Technology, 8707S) and β-III-tubulin (BioLegend, 801202), markers of mature neurons. Morphologically, the corrected cells displayed extensive neurite outgrowth, characteristic of neurons generated in this assay, whereas neurite formation was blunted in the unedited cells (Figure 2, F and G). Supplementing corrected NPCs with a membrane-permeable L-2HG ester (30 μM octyl-L-2HG) (10) completely reversed the effect of *L2HGDH* correction on total neurite length (Figure 2, F and G), indicating an L-2HG–dependent defect in neurite growth in the unedited cells.

RNA-seq was performed to further characterize the molecular differences between unedited and corrected cells. GSEA indicated enrichment of the REACTOME_NEURONAL_SYSTEM gene set in corrected compared with unedited neurons after 14 days of differentiation (Figure 2H); this was the highest scoring of all 3,917 gene sets by GSEA (Supplemental Figure 2A and Supplemental Table 1). To assess neuronal excitability, functional calcium imaging was performed in neuronal cultures following KCl stimulation (30). This assay revealed essentially no response in most unedited NPC-derived neurons, but prominent intracellular calcium increases in the corrected cells, indicative of improved neuronal activity (Figure 2I). Consistent with a delayed exit from the progenitor state, unedited neurons retained much higher levels of PAX6, a well-established marker of NPCs, compared with their corrected counterparts after 14 days of neuronal differentiation (Figure 2J).

To further resolve cell-type composition during neuronal differentiation in 3D, we performed scRNA-seq on day 45 cortical spheroids derived from unedited and corrected human iPSCs (hiPSCs). UMAP embedding revealed differences in cellular composition between conditions (Figure 2K). Unedited spheroids exhibited an increased proportion of immature, uncommitted neurons and a corresponding reduction in specified excitatory neurons relative to corrected spheroids (Figure 2, L and M), consistent with a block in neuronal specification.

Together, these findings highlight the impact of *L2HGDH* mutation and L-2HG accumulation on NPC proliferation, self-renewal, and neuronal differentiation. Our data demonstrate that CRISPR/Cas9-mediated correction of L2HGDH restores neuronal differentiation and function, suggesting the potential of this isogenic model to uncover the molecular mechanism by which L-2HG excess disrupts neuronal development.

### Depleting L-2HG enhances neuronal differentiation in L2HGDH-deficient NPCs.

**Figure 3:**
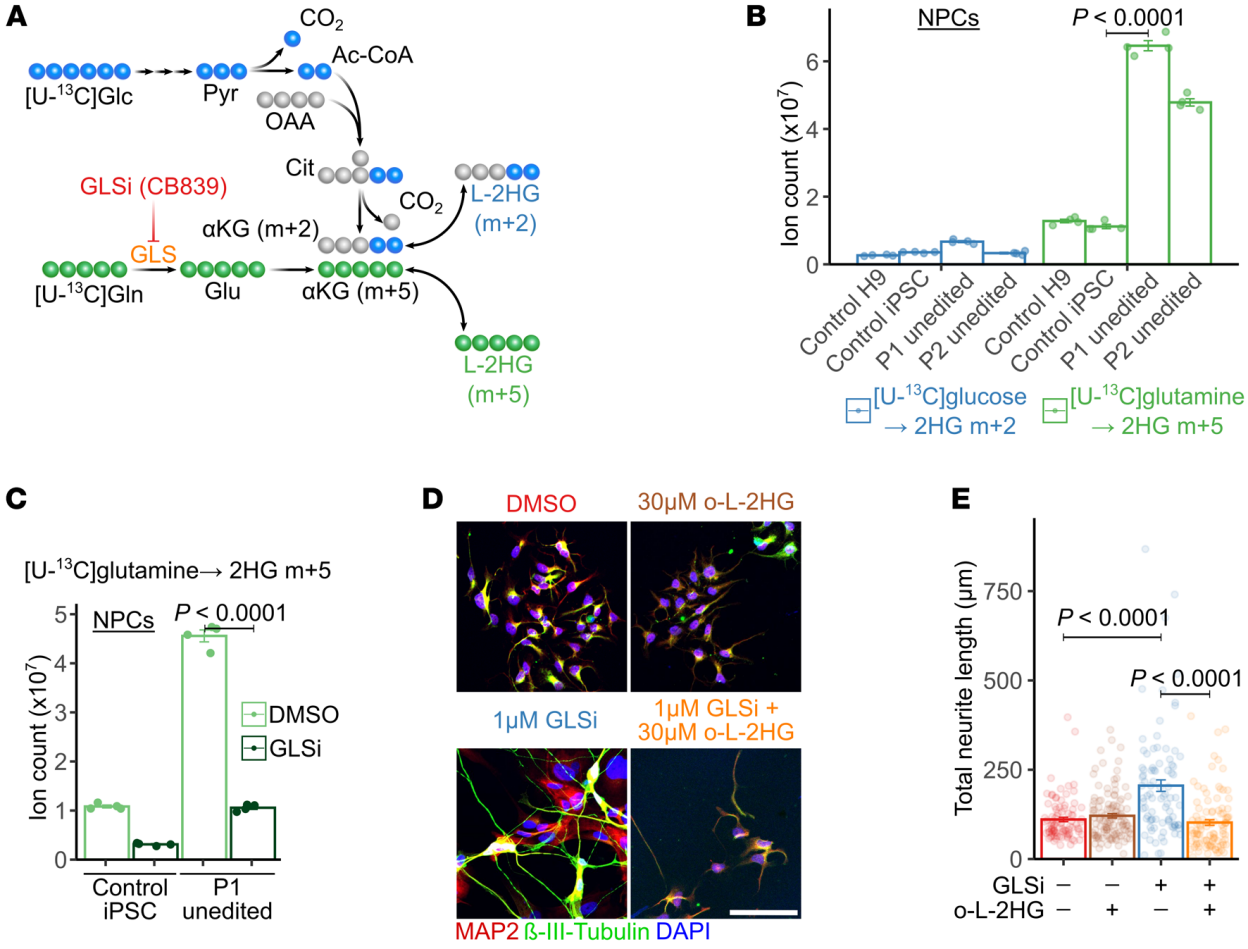
Suppressing L-2HG synthesis improves neurite formation in L2HGDH-deficient NPCs. (**A**) Schematic of ^13^C-labeling routes to L-2HG from [U-^13^C]glucose (blue) or [U-^13^C]glutamine (green) in NPCs. Glc, glucose; Pyr, pyruvate; Ac-CoA, acetyl-CoA; OAA, oxaloacetate; Cit, citrate; Gln, glutamine; GLS, glutaminase; GLSi, glutaminase inhibitor (CB-839); Glu, glutamate. (**B**) Ion counts of ^13^C-labeled 2HG isotopologues in NPCs derived from H9, control hiPSC, and patients 1 and 2 after 4 hours of labeling with [U-^13^C]glucose or [U-^13^C]glutamine. (**C**) Ion counts of ^13^C-labeled 2HG isotopologues in control hiPSC-derived and patient 1 NPCs cultured in [U-^13^C]glutamine and treated with DMSO or 1 μM CB-839 (GLSi) for 4 hours. (**D**) Immunofluorescence for MAP2 and β-III-tubulin in patient 1 NPCs after 14 days of neuronal differentiation, treated with DMSO, 30 μM octyl-L-2HG (o-L-2HG), 1 μM CB-839 (GLSi), or both. DAPI marks nuclei. Scale bar: 100 μm. (**E**) Quantification of total neurite length from cells in **D**, measured with the SNT plug-in in ImageJ. For **B** and **C**, ion counts were normalized to total ion current. Data were non-normally distributed (Shapiro-Wilk test), so statistical significance was assessed by Kruskal-Wallis test with Dunn’s post hoc correction (*n* = 4 per group). Significance was determined by 1-way ANOVA with Tukey’s HSD test for **E** (*n* = 90 for DMSO + DMSO, *n* = 116 for DMSO + o-L-2HG, *n* = 89 for GLSi + DMSO, *n* = 101 for GLSi + o-L-2HG). Data are shown as mean ± 1 SEM.

We next sought to test whether the defect in neuronal differentiation in L2HGDH-deficient NPCs could be reversed pharmacologically. We identified the predominant nutrient source of L-2HG by performing parallel stable isotope tracing experiments in medium containing [U-^13^C]glucose and unlabeled glutamine, or [U-^13^C]glutamine and unlabeled glucose. [U-^13^C]glucose is predicted to give rise to αKG and L-2HG m+2, while [U-^13^C]glutamine is predicted to give rise to αKG and L-2HG m+5 (Figure 3A; complete set of labeling data in Supplemental Tables 2 and 3). These isotope tracing experiments used a mass spectrometry approach that does not discriminate between L- and D-2HG, but note that L-2HG accounts for most of the total 2HG pool in L2HGDH-deficient cells (Figure 1H and Supplemental Figure 1E). In all cell lines examined, the abundance of ^13^C-labeled 2HG from glutamine exceeded the abundance from glucose, as expected if glutamine is the dominant source of αKG (Figure 3B). Unedited patient-derived NPCs cultured with [U-^13^C]glutamine had much more ^13^C-2HG than these same NPCs cultured in [U-^13^C]glucose or control NPCs cultured in [U-^13^C]glutamine (Figure 3B).

Given that glutaminase inhibition has been shown to reduce 2HG production and proliferation in IDH1-mutant glioma cells (31), we next investigated the effects of blocking glutamine metabolism by treating unedited NPCs with the glutaminase inhibitor (GLSi) CB-839 or DMSO. GLS inhibition reduced ^13^C-labeled L-2HG (m+5) derived from [U-^13^C]glutamine, indicating that GLS is required for glutamine-derived 2HG production in both control and L2HGDH-deficient NPCs in culture (Figure 3C). Consistent with inhibition of glutamine catabolism, treating unedited NPCs with CB-839 increased intracellular glutamine levels (Supplemental Figure 2B). Thus, the metabolic and functional effects of CB-839 in this system are unlikely to result from glutamine depletion, which can alter transcriptional programs (32).

To estimate the share of glutamine utilization resulting in L-2HG formation, we quantified total and labeled intra- and extracellular L-2HG after 4 hours of culture in medium containing [U-^13^C]glutamine and compared these values to net glutamine consumption. Unedited L2HGDH-deficient NPCs converted 0.46% ± 0.04% of consumed glutamine carbon to L-2HG during this interval, with similar abundances of L-2HG recovered from the intra- and extracellular space (Supplemental Figure 2C). Over the same period in the same cells, 16.85% ± 1.75% of consumed glutamine carbon appeared as secreted glutamate (Supplemental Figure 2D), indicating that production of L-2HG is a minor fate of glutamine carbon under these conditions. NPCs from both patients also displayed extensive labeling of TCA cycle intermediates. Labeling in citrate revealed similar m+4 and m+5 labeling, resulting from oxidation and reductive carboxylation of αKG, respectively (Supplemental Figure 2E).

Next, we evaluated whether CB-839 could enhance neuronal differentiation in L2HGDH-deficient NPCs. Immunofluorescence staining for the neuronal markers MAP2 and β-III-tubulin at day 14 of differentiation revealed approximately a doubling of total neurite length in unedited NPCs from patient 1 (Figure 3, D and E). This effect was completely reversed by supplementing the medium with 30 μM octyl-L-2HG (Figure 3, D and E). Octyl-L-2HG had no suppressive effect on neurite length in unedited cells treated with DMSO, where the intracellular L-2HG concentration is already high (Figure 3E). These results show that targeting cellular metabolism to reduce L-2HG levels can restore neuronal differentiation in L2HGDH-deficient NPCs.

### Increased MYC expression in L2HGDH-deficient NPCs and cortical spheroids.

**Figure 4:**
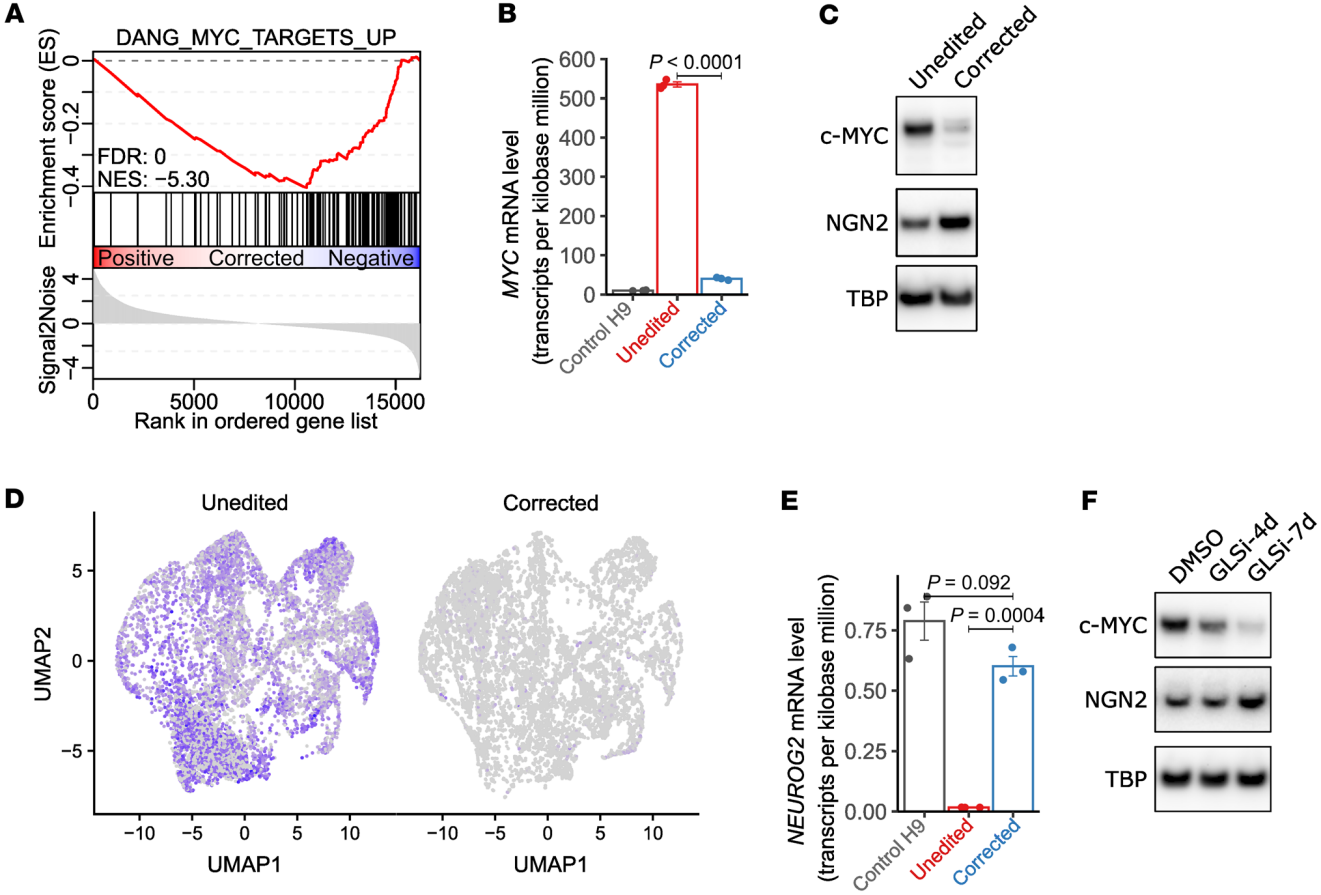
Increased *MYC* expression in L2HGDH-deficient NPCs and cortical spheroids. (**A**) GSEA mountain plot showing enrichment of DANG_MYC_TARGETS_UP gene set in unedited versus corrected patient 1 NPCs. (**B**) Quantification of *MYC* mRNA in control H9 NPCs, unedited patient 1 NPCs, and corrected patient 1 NPCs. mRNA levels were obtained from RNA-seq of biological triplicates and expressed as transcripts per kilobase million (TPM). (**C**) Immunoblot analysis of nuclear c-MYC and Neurogenin-2 in unedited and corrected patient 1 NPCs. TBP was used as a loading control for nuclear lysates. (**D**) UMAP heatmaps displaying *MYC* expression in scRNA-seq of unedited and corrected day 45 cortical spheroids. Color intensity (purple) indicates *MYC* transcript levels in individual cells. (**E**) Quantification of *NEUROG2* mRNA in control H9 NPCs, unedited patient 1 NPCs, and corrected patient 1 NPCs. mRNA levels were obtained from RNA-seq of biological triplicates and expressed as TPM. (**F**) Immunoblot analysis of nuclear c-MYC and Neurogenin-2 in patient 1 NPCs treated with DMSO or 1 μM CB-839 (GLSi) for 4 or 7 days. TBP served as a loading control for nuclear lysates. Significance was determined based on log-transformed TPM values using 1-way ANOVA followed by Tukey’s HSD test for **B** and **E**. Data are shown as mean ± 1 SEM of 3 biological replicates.

To broadly assess the molecular consequences of L2HGDH deficiency in NPCs and better understand the defect in neuronal differentiation, we conducted bulk RNA-seq in control, unedited, and corrected NPCs. Unbiased GSEA (33) identified the upregulation of MYC target genes in unedited NPCs (normalized enrichment score = –5.30) (Figure 4A). Among the 3,494 gene sets examined, 2 MYC target gene sets ranked within the top 10 and 4 ranked within the top 30 in unedited NPCs (Supplemental Figure 3A and Supplemental Table 4). RNA-seq also revealed a marked increase in *MYC* mRNA levels in unedited patient NPCs compared with both corrected NPCs and control H9 NPCs (Figure 4B). Immunoblot analysis of nuclear lysates revealed elevated c-MYC in unedited NPCs relative to corrected NPCs, supporting the transcriptional data (Figure 4C). Because these cells were generated with nonintegrating Sendai vectors, and Sendai clearance was verified by RT-PCR before downstream differentiation (Supplemental Figure 3B), persistent ectopic *MYC* from the reprogramming procedure does not explain elevated *MYC* in the NPCs.

We also evaluated MYC expression in day 45 cortical spheroids and observed increased *MYC* transcript levels in unedited spheroids relative to corrected spheroids, visualized by UMAP-based expression heatmaps (Figure 4D). *MYC* was upregulated in essentially all cell types in unedited spheroids, indicating widespread overexpression (Supplemental Figure 3C). Elevated *MYC* was observed in neurogenic radial glia, with marked upregulation in cycling intermediate progenitors and cycling gliogenic radial glia. This elevated expression persisted through both neuronal and glial lineages, including late neurogenic precursors, immature neurons, and gliogenic progenitors, suggesting that L-2HG–associated dysregulation of *MYC* occurs early in lineage commitment and is sustained across multiple descendant cell types. These data confirm that MYC overexpression extends beyond 2D NPC cultures to more complex 3D models of neurodevelopment.

We also evaluated *MYCN* given its overlap with *MYC* in neural progenitors (34). In 2D NPC cultures, *MYCN* transcript abundance showed only a modest increase in unedited cells relative to corrected cells (Supplemental Figure 3D), much smaller than the >12-fold induction observed for *MYC* (Figure 4B). Notably, *MYCN* levels in unedited L2HGDH-deficient NPCs remained lower than those in control H9 NPCs (Supplemental Figure 3D). In day 45 cortical spheroids, *MYCN* expression displayed the opposite pattern from *MYC*: several progenitor populations, including neurogenic radial glia, cycling gliogenic radial glia, and cycling intermediate progenitors, exhibited higher *MYCN* expression in corrected spheroids than in unedited spheroids (Supplemental Figure 3E). These findings indicate that *MYCN* is not the primary driver of altered proliferation and differentiation in L2HGDH-deficient NPCs and that *MYC* is selectively and disproportionately dysregulated by L-2HG.

Interestingly, we observed a concurrent decrease in Neurogenin-2, a key regulator of neuronal differentiation (35), in unedited cells (Figure 4E). To determine if pharmacological suppression of L-2HG production could mitigate *MYC* overexpression and restore *NEUROG2* (the gene encoding Neurogenin-2) expression, we treated unedited NPCs with CB-839 (GLSi) for 4 or 7 days. The drug reduced nuclear c-MYC levels in a time-dependent manner, accompanied by an increase in Neurogenin-2 expression (Figure 4F).

To validate the increased c-MYC levels in NPCs obtained from the embryonic mouse brain, we used a previously described *L2hgdh*-knockout mouse model (36). We dissected the dorsal telencephalon of WT and *L2hgdh^–/–^* E15.5 mouse embryos and cultured neurospheres ex vivo to obtain NPCs. To test whether L2HGDH deficiency impacts neuronal differentiation in this system, WT and *L2hgdh^–/–^* NPCs were differentiated into neurons for 3 weeks, fixed, and immunostained for MAP2 and β-III-tubulin. Quantification of neurite outgrowth revealed that *L2hgdh^–/–^* neurons exhibited shorter neurites than their WT counterparts (Supplemental Figure 3F). We then performed immunoblot analysis of nuclear c-MYC in WT and *L2hgdh^–/–^* NPCs. Consistent with human NPC data, *L2hgdh^–/–^* NPCs exhibited a modest increase in c-MYC protein levels compared with WT NPCs (Supplemental Figure 3, G and H).

### L2HGDH-deficient NPCs display enhanced activating histone methylation marks in MYC regulatory regions.

**Figure 5:**
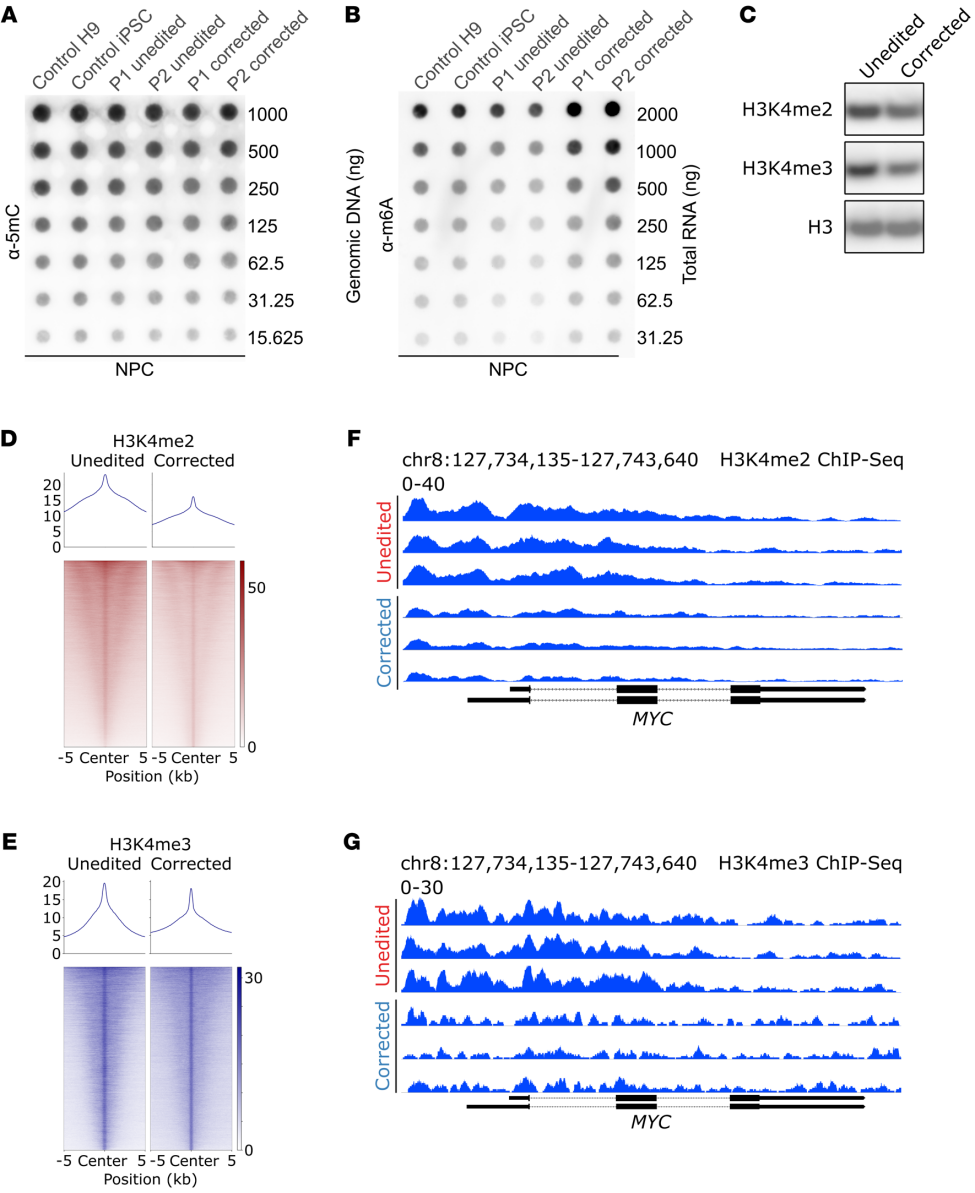
Increased activating histone methylation marks H3K4me2 and H3K4me3 at the *MYC* locus in L2HGDH-deficient NPCs. (**A**) DNA dot blot analysis of 5mC levels in NPCs, including control H9 NPCs, control iPSC-derived NPCs, unedited patient 1 NPCs, unedited patient 2 NPCs, corrected patient 1 NPCs, and corrected patient 2 NPCs. (**B**) m6A dot blot analysis of total RNA in NPCs, including control H9 NPCs, control iPSC-derived NPCs, unedited patient 1 NPCs, unedited patient 2 NPCs, corrected patient 1 NPCs, and corrected patient 2 NPCs. (**C**) Immunoblot analysis of activating histone markers H3K4me2 and H3K4me3 levels in unedited and corrected patient 1 NPCs. Histone H3 was used as a loading control for histone lysates. (**D** and **E**) ChIP-seq profiles of H3K4me2 (**D**) and H3K4me3 (**E**) in unedited and corrected patient 1 NPCs. ChIP-seq signals were plotted over center peaks (±5 kb from peak center) identified in corrected patient 1 NPCs. Sites were sorted by the ChIP-seq signal intensity from corrected patient 1 NPCs. (**F** and **G**) Representative ChIP-seq tracks showing H3K4me2 (**F**) and H3K4me3 (**G**) enrichment at the *MYC* locus in unedited and corrected patient 1 NPCs.

Given the potentially broad roles of L-2HG and D-2HG in blocking epigenetic demethylases that use αKG as a cofactor, we compared several aspects of the epigenetic landscape between unedited and corrected NPCs. We did not observe an increase in global DNA methylation at cytosine residues (5mC; Figure 5A) or in global N^6^-methyladenosine in mRNA (m6A; Figure 5B) in NPCs with L-2HG accumulation. Rather, m6A methylation was somewhat higher in corrected NPCs compared with unedited NPCs. Thus, we did not observe global suppression of 5mC and m6A demethylation in these L2HGDH-deficient cells, despite 2HG’s ability to block TET DNA demethylases and the RNA demethylases ALKBH5 and FTO in other contexts (37, 38). We estimated the total (i.e., noncompartmentalized) intracellular concentration of L-2HG in L2HGDH-deficient NPCs to be 1,425 ± 75 μM (mean ± SEM). This concentration is several fold higher than the reported IC_50_ values for most histone lysine demethylases (KDMs) (39, 40) and somewhat below the reported IC_50_ for TET2 (41), which may explain why L-2HG accumulation apparently does not impact global DNA methylation in L2HGDH-deficient NPCs.

We next examined histone methylation, focusing on marks associated with active transcription because of the observation that *MYC* expression is enhanced in L2HGDH-deficient NPCs. Methylation of histone H3, lysine residue 4 (H3K4) is associated with gene activation in development (42). Immunoblot analysis revealed reduced dimethylated and trimethylated H3K4 (H3K4me2 and H3K4me3) in corrected NPCs compared with unedited cells (Figure 5C). To characterize the genomic distribution of these marks, we performed ChIP-seq for H3K4me2 and H3K4me3. Aggregated ChIP-seq signals plotted over peak centers (±5 kb) demonstrated globally increased enrichment of both H3K4me2 and H3K4me3 in unedited NPCs relative to corrected NPCs (Figure 5, D and E). This widespread elevation suggests that L2HGDH deficiency contributes to broad epigenetic reprogramming in NPCs via changes in H3K4 methylation. Unbiased differential binding analysis of H3K4me2 and H3K4me3 ChIP-seq data further confirmed that both marks were prominently increased at *MYC* regulatory regions and ranked among the most hypermethylated sites in unedited NPCs compared with corrected NPCs (Supplemental Figure 4A and Supplemental Tables 5 and 6). Focusing on the *MYC* locus, representative ChIP-seq tracks highlighted pronounced enrichment of H3K4me2 and H3K4me3 in unedited NPCs compared with corrected cells (Figure 5, F and G). When all enriched genomic regions were mapped to their associated genes, unbiased Kyoto Encyclopedia of Genes and Genomes overrepresentation analysis identified “cell cycle” as the top enriched pathway for both activating H3K4 methylation marks in unedited NPCs (Supplemental Figure 4, B and C, and Supplemental Tables 7 and 8), aligning with the hyperproliferative phenotype observed in these cells.

### KDM5 inhibition recapitulates the effects of L-2HG on H3K4 methylation and neuronal differentiation.

KDM5 family enzymes are the primary demethylases responsible for removing H3K4 methylation (43) and are greatly inhibited by L-2HG (10). Given that L2HGDH-deficient NPCs exhibit increased H3K4me2 and H3K4me3 levels, we sought to determine whether pharmacological inhibition of KDM5 would mimic the effects of L-2HG accumulation and impair neuronal differentiation. To test this, we treated corrected patient 1 NPCs with the selective KDM5 inhibitor C70 (44) (25 μM) for 14 days. Immunoblot analysis confirmed that KDM5 inhibition increased H3K4me2 and H3K4me3 levels compared with DMSO-treated controls, supporting KDM5’s role in H3K4 demethylation in these cells (Supplemental Figure 4D). ChIP-seq analysis further revealed that KDM5 inhibition resulted in a genome-wide increase in H3K4me2 and H3K4me3 levels (Supplemental Figure 4, E and F). Representative ChIP-seq tracks confirmed that C70-treated NPCs exhibited higher H3K4me2 and H3K4me3 deposition at the *MYC* promoter compared with DMSO-treated controls (Supplemental Figure 4, G and H).

Given that L-2HG–mediated *MYC* overexpression is associated with impaired neuronal differentiation, we next examined whether KDM5 inhibition influenced *MYC*. qPCR analysis revealed a moderate increase in *MYC* mRNA abundance in C70-treated NPCs (Supplemental Figure 4I). Similarly, immunoblot analysis of nuclear lysates revealed that C70 treatment resulted in modestly increased c-MYC expression relative to DMSO-treated NPCs (Supplemental Figure 4J). Finally, to assess the functional impact of KDM5 inhibition on neuronal differentiation, we quantified neurite outgrowth following treatment. Consistent with the effects of L-2HG accumulation, C70-treated NPCs exhibited reduced neurite length compared with controls, indicating that KDM5 inhibition negatively impacts neuronal differentiation (Supplemental Figure 4K).

These findings indicate that inhibition of KDM5, like L-2HG accumulation, increases H3K4 methylation, increases *MYC* expression, and impairs neurite length. This supports the idea that L-2HG may exert its effects, at least in part, through inhibition of KDM5 demethylases.

### L2HGDH-deficient NPCs exhibit genome-wide gains of repressive histone methylation that spare the MYC locus.

To assess whether L2HGDH deficiency also affects repressive chromatin, we performed ChIP-seq for H3K9me3 and H3K27me3 in unedited and corrected NPCs. Both marks were globally increased in L2HGDH-deficient cells (Supplemental Figure 5, A and B). However, neither H3K9me3 nor H3K27me3 was elevated across the *MYC* locus (Supplemental Figure 5, C and D). These data indicate that L-2HG drives widespread accumulation of repressive histone methylation marks despite enhanced *MYC* expression.

### c-MYC depletion restores neuronal differentiation in L2HGDH-deficient NPCs.

**Figure 6:**
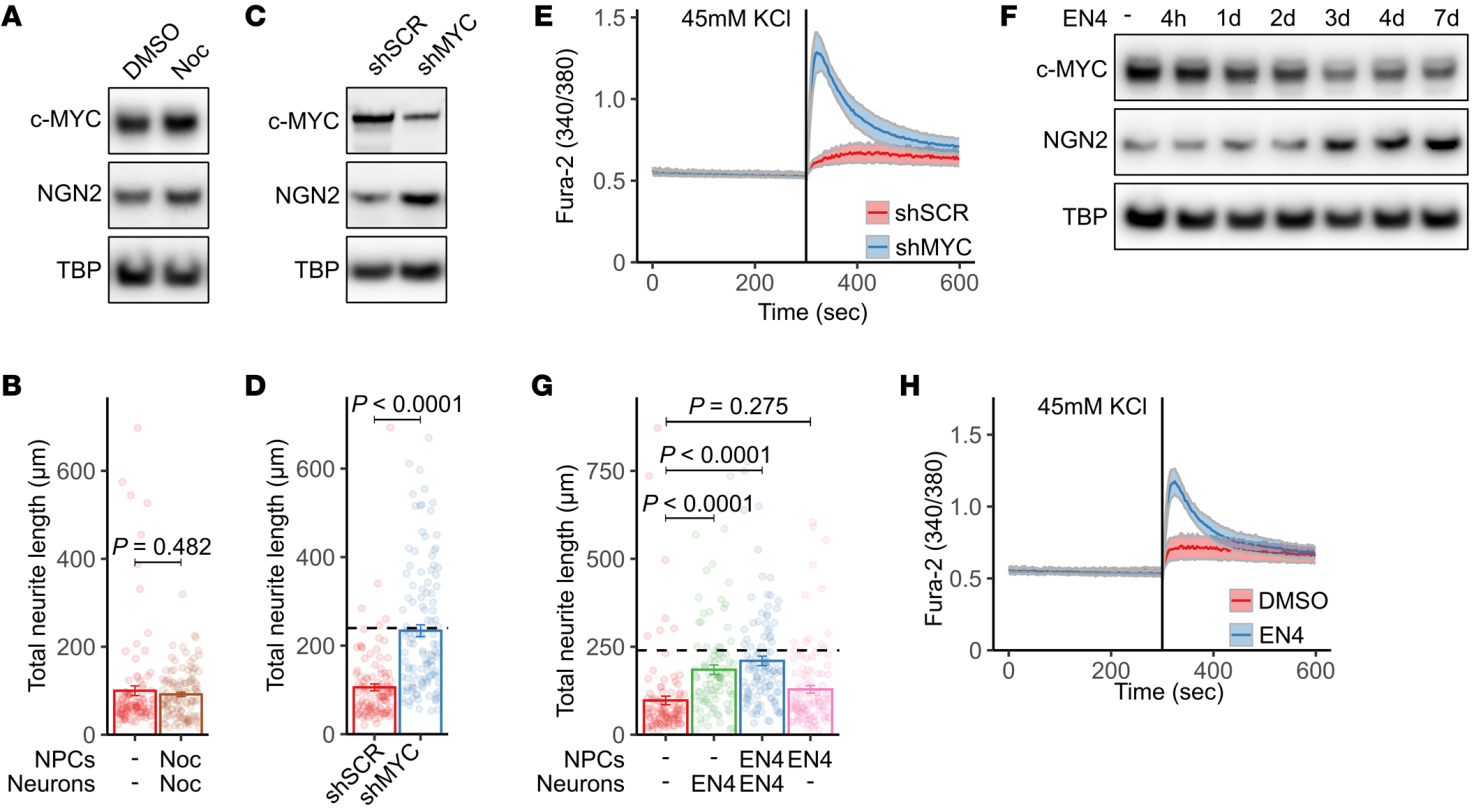
c-MYC depletion restores neuronal differentiation in L2HGDH-deficient NPCs. (**A**) Immunoblot of nuclear c-MYC and Neurogenin-2 (NGN2) in unedited patient 1 NPCs treated with DMSO or 20 μM nocodazole (Noc) for 7 days. TBP was used as a loading control. (**B**) Quantification of neurite lengths in patient 1 NPCs treated with DMSO or 20 μM Noc at the NPC stage and throughout 14 day neuronal differentiation. (**C**) Immunoblot of nuclear c-MYC and Neurogenin-2 in patient 1 NPCs transduced with lentivirus expressing control shRNA (shSCR) or shMYC. (**D**) Quantification of neurite lengths in patient 1 NPCs transduced with shSCR or shMYC. The dashed line denotes the mean neurite length in corrected neurons. (**E**) Representative recordings of intracellular Ca^2+^ dynamics in neurons transduced with shSCR (*n* = 29) or shMYC (*n* = 24), captured every 10 seconds using Fura-2 ratio imaging during 45 mM KCl application. Neurons were differentiated for 47 days. (**F**) Immunoblot of nuclear c-MYC and Neurogenin-2 in patient 1 NPCs treated with DMSO or 50 μM EN4 for the indicated durations. (**G**) Quantification of neurite lengths in patient 1 NPCs treated with DMSO, EN4 throughout differentiation, EN4 at the NPC stage and throughout differentiation, or EN4 only at the NPC stage. The dashed line indicates the corrected neuron mean. (**H**) Representative Ca^2+^ recordings in neurons treated with DMSO (*n* = 28) or 50 μM EN4 (*n* = 26), acquired as in **E**. For **B**, **D**, and **G**, neurite lengths were measured using the SNT plug-in in ImageJ. Data are shown as mean ± 1 SEM. Significance was determined by unpaired 2-tailed Student’s *t* test for **B** and **D** or 1-way ANOVA with Tukey’s HSD test for **G**.

Although correcting mutant *L2HGDH* alleles reduced H3K4 methylation at hundreds of genomic loci, the prominent effect on the *MYC* locus and the broad roles of *MYC* in cell proliferation and differentiation suggested that *MYC* expression could be sufficient to explain the effects of L2HGDH deficiency. We first ruled out the possibility that impaired neurite length was a nonspecific response to enhanced proliferation. Unedited NPCs were treated with the antimitotic agent nocodazole at a concentration that maintained NPC viability throughout the treatment duration (Supplemental Figure 6A). As expected, nocodazole suppressed NPC proliferation (Supplemental Figure 6B) but had no impact on the expression of c-MYC or Neurogenin-2 (Figure 6A). Similarly, neurite length was not enhanced by nocodazole (Figure 6B). This indicates that cell cycle modulation is insufficient to restore neuronal differentiation in L2HGDH-deficient NPCs.

Next, we used lentiviral shRNA to suppress *MYC* expression. Immunoblot analysis confirmed reduced c-MYC in NPCs transduced with *MYC*-targeting shRNA (shMYC) lentivirus compared with a scrambled control RNA (shSCR; Figure 6C). This reduction was accompanied by an increase in Neurogenin-2. Quantification of neurite outgrowth showed that shMYC doubled the average total neurite length in unedited NPCs compared with shSCR controls (Figure 6D). To evaluate whether c-MYC depletion also enhanced functional neuronal properties, we performed calcium imaging on neurons transduced with shMYC. Compared with neurons transduced with shSCR, c-MYC–depleted neurons exhibited markedly increased calcium influx in response to KCl stimulation, indicating enhanced excitability (Figure 6E). This functional improvement mirrors the calcium response observed in corrected neurons (Figure 2I), further supporting the role of c-MYC downregulation in rescuing neuronal function.

To pharmacologically inhibit c-MYC, we assessed small-molecule compounds for acceptable on-target activity and tolerability in NPCs. MYCi975 (45) caused marked cytotoxicity in human NPCs (Supplemental Figure 6C), precluding mechanistic assays at nonlethal doses. 10074-G5 (46), a c-MYC/MAX interaction inhibitor, was better tolerated but did not reduce nuclear c-MYC by immunoblot at the doses tested (Supplemental Figure 6, C and D). We also evaluated EN4, which targets cysteine 171 of c-MYC within an intrinsically disordered region, reducing c-MYC and MAX thermal stability and inhibiting MYC transcriptional activity (47). EN4 reduced c-MYC protein abundance at tolerable doses (Figure 6F and Supplemental Figure 6C). Suppression of c-MYC abundance was time dependent and correlated with increased Neurogenin-2 expression (Figure 6F). Quantification of neurite outgrowth in NPCs subjected to EN4 demonstrated that continuous EN4 treatment during both the NPC and neuronal differentiation stages enhanced total neurite length, nearly to the same degree as correcting the *L2HGDH* allele (Figure 6G). In contrast, EN4 treatment limited to the NPC stage had no effect compared with controls, indicating that sustained inhibition of c-MYC is necessary to reverse differentiation defects in this model (Figure 6G). To assess functional improvement, we measured neuronal calcium responses using Fura-2 ratio imaging. EN4-treated neurons exhibited enhanced intracellular calcium influx in response to KCl stimulation compared with DMSO-treated controls (Figure 6H), indicating that pharmacologic suppression of c-MYC not only improves neuronal morphology but also restores neuronal function in L2HGDH-deficient cells.

Because *MYC* can activate *L2HGDH* transcription in *MYC*-amplified cancers (48), we also tested whether *MYC* suppression alters expression of either *L2HGDH* or *D2HGDH*. In unedited NPCs, shMYC had no effect on *L2HGDH* mRNA, but modestly reduced *D2HGDH* mRNA (Supplemental Figure 6, E and F). Thus, in this context, c-MYC inhibition does not modulate *L2HGDH* expression.

## Discussion

Many IEMs manifesting in the CNS cause catastrophic episodes of metabolic decompensation, frequently early in life, leading to acute damage to the brain in the setting of acidosis, hyperammonemia, hypoglycemia, and other complications of severe, systemic metabolic dysfunction. In contrast, the cerebral organic acidemias, including L2HGDH deficiency, are often characterized by a later or more insidious onset, progressive neurological dysfunction, and macrocephaly (49). The pathophysiological mechanisms of neurological dysfunction in cerebral organic acidemias are not completely understood, and the striking clinical differences from decompensating disorders raise the possibility of mechanisms beyond alterations in intermediary metabolism. The chronic nature of L2HGDH deficiency implies that its effects accumulate over time, possibly due to persistent epigenetic and transcriptional abnormalities induced by L-2HG accumulation (50, 51). The association of L2HGDH deficiency with pediatric tumors, particularly in the brain, also implies dysfunction of terminal differentiation programs in this disease. Our findings support this model by demonstrating that L-2HG inhibits demethylation of a subset of histone marks in NPCs, induces widespread changes in gene expression, and suppresses differentiation into neurons. This results in an overabundance of immature cells and a depletion of excitatory neurons in cortical spheroids.

In our models, L2HGDH deficiency promoted NPC proliferation and self-renewal at the expense of neuronal differentiation. A pivotal effect of L-2HG accumulation is the activation of *MYC* expression. c-MYC has myriad effects in support of cell proliferation and has been reported to contribute to NPC identity (52–54) and suppress neuronal differentiation (34, 55). c-MYC’s involvement in the phenotype of L2HGDH-deficient NPCs is further supported by its functions in glioma, including glioma stem cell maintenance (56, 57). Substantial evidence connects *MYC* specifically to the pathophysiology of IDH1/2-mutant gliomas, with elevated nuclear c-MYC expression in most of these tumors (58). The *MYC* locus has been identified as the most frequently amplified genomic region during glioma malignant progression, with such amplification correlating with increased tumor aggressiveness (59). Furthermore, rs55705857, a noncoding single-nucleotide polymorphism, is associated with a 6-fold increased risk of developing gliomas with mutations in *IDH1* or *IDH2* (60). This locus lies within a brain-specific enhancer, with the risk allele promoting interaction with the *MYC* locus and driving *MYC* expression (61). Engineering mice to contain the orthologous risk allele is sufficient to increase the penetrance and decrease the latency of IDH1-mutant gliomas (61).

L-2HG can accumulate under hypoxic conditions via LDH/MDH-dependent reduction of αKG (16, 17). In *Drosophila*, developmental elevations of L-2HG modulate chromatin and gene expression (22, 62). Our finding that elevated L-2HG activates *MYC* expression suggests a mechanistic bridge to perinatal hypoxic brain injury, such as hypoxic-ischemic encephalopathy and periventricular leukomalacia, in which transient L-2HG elevations could bias neural progenitor programs similarly to the L2HGDH-deficient state (63, 64).

Beyond neurogenesis, single-cell analyses showed *MYC* upregulation in gliogenic radial glia and early glial intermediates in L2HGDH-deficient cultures. Prior studies indicated that c-MYC controls oligodendrocyte progenitor cell proliferation and the transition to differentiation (65, 66), and L-2HGDH–deficient mice and patients exhibit white matter and oligodendroglial pathology (67, 68) with increased glioma risk (69, 70). These data support a model in which an L-2HG/KDM5/*MYC* axis perturbs the timing and maturation of astrocytes and oligodendrocytes, providing a glial contribution to L-2HGA pathogenesis. In L2HGDH-deficient NPCs, L-2HG broadly inhibits αKG-dependent histone demethylases, producing genome-wide increases in H3K9me3 and H3K27me3, while selectively elevating H3K4me2/3 at the *MYC* promoter. This locus-specific pattern is consistent with the notion that H3K4me3 stabilizes active transcription, facilitates RNA polymerase II pause-release, and resists Polycomb repression (71, 72). ChIP-seq revealed thousands of genomic sites with altered H3K4me2 and H3K4me3 between unedited and corrected NPCs, including increased abundance in the *MYC* regulatory region. These marks likely resulted from suppression of KDM5 demethylases because these enzymes are potently inhibited by L-2HG and because treating corrected cells with a KDM5 inhibitor phenocopied the effect of L2HGDH deficiency on these marks. Despite the widespread changes of H3K4me2 and H3K4me3, genetically or pharmacologically suppressing a single gene – *MYC* – was sufficient to induce levels of neurite formation similar to the effect of editing mutant *L2HGDH* to WT. It is possible that other processes beyond H3K4 methylation regulate *MYC* in these models because KDM5 inhibition had a large effect on H3K4 methylation but more modest effects on *MYC* expression and neurite formation. DNA demethylation (10) and RNA demethylation (37, 73) also involve αKG-dependent dioxygenases and can be blocked by 2HG in some systems. We did not observe global differences in DNA or RNA methylation between unedited and corrected cells, but we cannot rule out localized effects that might impact *MYC*. Nevertheless, our data indicate that high levels of L-2HG promote a chromatin state that favors *MYC* transcription, and it is remarkable that neuronal differentiation could be so effectively stimulated by manipulating this single gene among the thousands of loci altered by L2HGDH deficiency.

Because human L2HGDH deficiency involves progressive, postnatal neurological compromise, there may be opportunities to block disease progression using drugs that interrupt the biological effects of high L-2HG. Along these lines, the chronic, developmental nature of L2HGDH deficiency places the therapeutic focus on long-term management, which perhaps could include therapies aimed at suppressing neural stem cell self-renewal or stimulating neuronal differentiation. We report 2 different drugs that stimulate neurite formation in L2HGDH-deficient NPCs: EN4, a small molecule that reduces c-MYC stability (47), and CB-839, a GLSi. The mechanism by which CB-839 stimulates neurite formation is through depletion of L-2HG because glutamine is the predominant source of αKG in these cultured cells. It should be emphasized that this is likely not the case in the brain, which supplies the TCA cycle with other fuels, particularly pyruvate. But the conceptual importance of these therapeutic experiments is that the differentiation defect in NPCs can be reversed using multiple pharmacological approaches. Future studies should explore the efficacy of postnatal therapeutic interventions in preclinical models, particularly in targeting epigenetic and transcriptional dysregulation, to determine whether early treatment can alter the disease course and improve neurological function.

Both L-2HG and D-2HG have been demonstrated to interfere with epigenetic reprogramming by altering the function of αKG-dependent dioxygenases. This would seem to imply that the 2 enantiomers have largely redundant biological effects. However, in vitro studies have revealed markedly different inhibitory potencies of these molecules on specific dioxygenases, with distinct *K_I_* values reported for enzymes such as the TET family of hydroxylases and the KDM family of histone demethylases. These biochemical differences indicate that their downstream consequences are not identical and that the relative sensitivity of epigenetic regulators to each enantiomer could drive divergent biological outcomes (41). In this context, the disparate clinical and developmental phenotypes of diseases associated with high L-2HG or D-2HG are intriguing. L-2HG elevation in the setting of germline *L2HGDH* mutations increases the risk of pediatric tumors in the brain and elsewhere. In adults, D-2HG elevation in the setting of somatic mutations in *IDH1* or *IDH2* also promotes tumorigenesis in the brain and some other organs. Yet, children with diseases that cause systemic elevations of D-2HG have not been reported to have tumors in the brain. Isolated D-2HGA can be caused by biallelic mutations in *D2HGDH* or by monoallelic gain-of-function mutations in *IDH1/2*. These patients have neurodevelopmental disability and, in states of very high D-2HG, cardiomyopathy. Myeloid leukemias were reported in 2 children, one of whom also had a complex chromosomal rearrangement (74, 75), and other children have had benign tumors (76). It is unknown why these patients seem not to have a high risk of brain tumors despite the clear association between D-2HG and gliomas in adults. This suggests that the impact of L-2HG and D-2HG depends on the lineage and perhaps the developmental stage. Isogenic, patient-derived iPSC models that can be differentiated into various cell types should be useful to decode relationships between dioxygenase expression, epigenetics, lineage specification and the 2 enantiomers of 2HG.

## Methods

### Sex as a biological variable.

The iPSC lines were generated from 2 female patients with L2HGDH deficiency. Sex-based differences were not a variable in the design or interpretation of experiments involving cells from the patients. In mouse studies, E15.5 embryos were used without sex determination.

### Human fibroblast culture.

Human fibroblasts were derived from skin biopsies (patients 1 and 2; Karachi, Pakistan; collected under an IRB-approved protocol with written informed consent) or obtained from commercial sources (Lonza, CC-2509). Fibroblasts were cultured in DMEM (Sigma-Aldrich, D5796) supplemented with 10% FBS, 1% nonessential amino acids (NEAAs; Gibco, 11140-050), and 1% penicillin-streptomycin (Pen-Strep). Cultures were incubated at 37°C in a humidified atmosphere containing 5% CO_2_. Fibroblasts were regularly tested for mycoplasma contamination and maintained for no more than 10 passages. For experiments, fibroblasts were allowed to adhere overnight before downstream applications.

### D-2HG and L-2HG quantitation.

Metabolites were extracted using an 80% methanol–water solution. The resulting supernatant was dried in a SpeedVac, and the pellet was resuspended in a diacetyl-l-tartaric anhydride derivatization solution (90 μL of 50 mg/mL diacetyl-l-tartaric anhydride in freshly prepared 80% acetonitrile/20% acetic acid; Acros Organics). [U-^13^C]D/L-2HG (Cambridge Isotope Laboratories; 10 ng in 10 μL acetonitrile) was added as an internal standard. The mixture was sonicated, incubated at 75°C for 30 minutes, and cooled to room temperature before centrifugation to collect the supernatant. The supernatant was dried in a SpeedVac and reconstituted in 100 μL of 1.5 mmol/L ammonium formate aqueous solution with 10% acetonitrile for liquid chromatography–mass spectrometry analysis as described (51).

### iPSC generation.

These experiments were conducted in accordance with guidelines established by the University of Texas Southwestern Medical Center (UTSW) Stem Cell Research Oversight Committee. iPSCs were generated using the CytoTune-iPS 2.0 Sendai Reprogramming Kit (Thermo Fisher Scientific), which delivers OCT4, KLF4, SOX2, and c-MYC via a nonintegrating Sendai virus, allowing for footprint-free reprogramming. Fibroblasts were cultured in DMEM with 10% FBS, 1% NEAAs, 1% penicillin-streptomycin, and 0.1 mM β-mercaptoethanol until 30%–60% confluency. Cells were transduced with the Sendai virus vectors at a multiplicity of infection of 5:5:3 (KOS: MYC: KLF4). After 24 hours, the medium was replaced with fresh fibroblast medium, and cells were cultured for 6 days before passaging onto irradiated mouse embryonic fibroblast feeder layers (GlobalStem) in hiPSC medium (DMEM/F-12, 20% knockout serum replacement, 1% NEAAs, 0.1 mM β-mercaptoethanol, and 10 ng/mL bFGF). Media were replaced daily, and colonies with embryonic stem cell–like morphology emerged within 12–21 days.

For each patient, 8 clonal iPSC lines were derived and 2 were further characterized. Reprogramming was confirmed through immunofluorescence for OCT4 (Cell Signaling Technology, 94310), SOX2 (Cell Signaling Technology, 3579S), NANOG (Cell Signaling Technology, 99399), TRA-1-60 (Cell Signaling Technology, 61220), and TRA-1-81 (Cell Signaling Technology, 83321). Clearance of exogenous *MYC* was verified by RT-PCR at passage ≥10 using primers specific to the Sendai-derived *MYC* (forward 5′-TAACTGACTAGCAGGCTTGTCG-3′; reverse 5′-TCCACATACAGTCCTGGATGATGATG-3′). RT-PCR conditions were as follows: 35 cycles; 95°C denaturation, 55°C annealing, and 72°C extension.

### iPSC CRISPR/Cas9 genome editing.

PX458 cloning with single-step digestion-ligation was used to generate a Cas9-sgRNA expression plasmid containing GFP, with the sgRNA targeting the *L2HGDH* c.829C>T mutation. Feeder-free iPSCs were dissociated into single cells using Accutase (Thermo Fisher Scientific). The electroporation mixture contained the PX458 plasmid and the ssODN repair template, resuspended to 100 μL per electroporation. Electroporation used the Amaxa Human Stem Cell Nucleofector Kit 1 (Lonza) with a Lonza Nucleofector device under program B-016. Immediately after nucleofection, cells were plated in mTeSR1 medium supplemented with 10 μM ROCK inhibitor. 48 hours later, GFP^+^ cells were isolated via FACS, seeded at clonal density, and expanded in mTeSR1 medium on Matrigel-coated plates. Colonies were picked and expanded for genotyping. RFLP analysis was performed using *Btg*I, and clones exhibiting a digestion pattern consistent with successful editing were validated by Sanger sequencing.

### NPC generation.

NPCs were generated using a dual-SMAD inhibition neural specification protocol (26). Feeder-free iPSCs were cultured in mTeSR1 medium on Matrigel-coated plates until approximately 70%–80% confluency, then transitioned to N2/B27 neural induction medium supplemented with 0.1 μM LDN193189 and 10 μM SB431542. On day 14, neural rosettes were isolated and transferred to poly-l-ornithine/laminin–coated plates in NPC expansion medium containing N2, B27, and 20 ng/mL FGF2. Medium was changed every other day, and cells were passaged with Accutase. NPC identity was tested by immunocytochemistry for NESTIN (Abcam, ab22035) and SOX2 (Cell Signaling Technology, 3579S).

### Cortical spheroid generation and measurement.

Cortical spheroids were generated using an established protocol (27). NPCs were cultured in low-attachment plates with cortical spheroid differentiation medium supplemented with LDN193189 and SB431542. After 14 days, spheroids were transferred to neural maturation medium containing BDNF and NT3. Spheroids were imaged using a Zeiss SteREO Discovery V.8 stereomicroscope, and ImageJ (NIH) was used to determine size.

### Clonogenicity assay.

Clonogenicity was assessed using a modified neural colony-forming cell assay (28). NPCs were dissociated into single cells, passed through a 40 μm cell strainer, and cultured for 21 days. On day 21, colonies were imaged by microscopy and counted using ImageJ.

### Neuronal differentiation and neurite imaging and quantification.

NPCs were dissociated into a single-cell suspension using Accutase, followed by neutralization with NPC medium and centrifugation at 300*g* for 5 minutes. The pellet was resuspended in neuronal differentiation medium (DMEM/F12 with 1× N2 supplement, 1× B27 supplement [without retinoic acid], 20 ng/mL BDNF [Peprotech], 20 ng/mL GDNF [Peprotech], 1 mM dibutyryl-cAMP [Sigma-Aldrich], 200 nM ascorbic acid [Sigma-Aldrich], and 1 μg/mL laminin [Gibco]), and cells were plated onto PLO/laminin-coated 6-well plates at 200,000 cells per well. The medium was changed every 3 days. On day 14, cells were immunostained for MAP2 and β-III-tubulin, and confocal images were acquired using a Nikon CSU-W1 spinning disk confocal microscope with SoRa. Neurite outgrowth was analyzed using the SNT (simple neurite tracer) plug-in in ImageJ (Fiji).

### Isolation and culture of mouse NPCs.

NPCs were isolated from the telencephalon of E15.5 mouse embryos as described (77). Neurosphere formation was typically observed within 3–5 days of obtaining NPCs. For differentiation, neurospheres were plated onto poly-d-lysine/laminin–coated 8-well chamber slides and maintained in NEP basal medium supplemented with 2% heat-inactivated horse serum (Gibco, 26050070) for 2–3 days, with daily medium changes. For neurite analysis, cultures were fixed in 4% paraformaldehyde and immunostained for neuronal markers.

### Ca^2+^ imaging.

Ca^2+^ imaging experiments were performed on neuronal cultures at day 47 following established protocols (78). Neurons were loaded with 5 μM Fura-2 AM (Molecular Probes) and incubated for 30 minutes at 37°C in Tyrode solution (150 mM NaCl, 4 mM KCl, 1.5 mM CaCl_2_, 2 mM MgCl_2_, 10 mM glucose, and 10 mM HEPES, pH 7.4). Coverslips containing neurons were mounted onto a recording/perfusion chamber (RC-26G, Warner Instruments) positioned on the movable stage of an Olympus IX-70 inverted microscope. Neuronal activity was assessed by stimulating with 45 mM KCl in Tyrode solution and detecting Ca^2+^ responses via excitation at 340 and 380 nm using a DeltaRAM illuminator (PTI) with a Fura-2 dichroic filter cube (Chroma Technologies) and a ×60 UV-grade, oil-immersion objective (Olympus). Emitted fluorescence was captured using an IC-300 camera (PTI), and images were digitized and analyzed using ImageMaster Pro software (PTI).

### Histone extraction.

Histones were extracted from cultured cells following an acid extraction to preserve posttranslational modifications. Cells were grown in 15 cm dishes until approximately 70% confluency, then washed twice with ice-cold PBS with 5 mM sodium butyrate. Cells were collected by scraping in 1 mL of PBS with sodium butyrate, followed by centrifugation at 1,000*g* for 5 minutes at 4°C. The pellet was resuspended in Triton extraction buffer (TEB; PBS supplemented with 0.5% Triton X-100 [v/v], 2 mM PMSF, and 0.02% [w/v] sodium azide) at a density of 1 × 10^7^ cells/mL. Cells were lysed on ice for 10 minutes, then centrifuged at 1,000*g* for 10 minutes at 4°C. The nuclear pellet was washed with 0.5× volume of TEB, followed by centrifugation at 1,000*g* for 10 minutes at 4°C. The pellet was resuspended in 0.2 N HCl at a final concentration of 4 × 10^7^ cells/mL and incubated overnight at 4°C on a rotating platform. The samples were then centrifuged at 1,000*g* for 10 minutes at 4°C, and the histones were collected in the supernatant. Protein concentration was determined using the Bradford assay, and aliquots were stored at –80°C for long-term use. Western blots used 2 μg of histone extract. Histone H3 was used as a loading control (Cell Signaling Technology, 4499).

### DNA dot blot analysis.

DNA dot blot analysis was performed using a Bio-Dot Microfiltration Apparatus (Bio-Rad) to assess global DNA methylation. Genomic DNA was isolated, quantified (Qubit), and fragmented to approximately 200–500 bp (Covaris, ME220). Fragmented DNA (50 ng/μL) was denatured (2× buffer: 200 mM NaOH and 20 mM EDTA; 95°C, 10 minutes), chilled on ice, neutralized with 20× SSC, adjusted to 500 μL, and diluted. DNA dilutions (100 μL/well) were applied to prewetted Zeta-Probe membranes (2× SSC) using vacuum and a UV crosslinker (1,200 J/m^2^; Stratagene, Stratalinker). Membranes were blocked and incubated with primary antibodies overnight (4°C) (Cell Signaling Technology, 28692), followed by HRP-conjugated secondary antibodies (Cell Signaling Technology 7074), ECL development, and imaging (GE HealthCare imager).

### RNA dot blot analysis.

RNA dot blot analysis was performed using a Bio-Dot Microfiltration Apparatus to assess global RNA modifications. Total RNA was extracted, quantified (Qubit), and assessed for integrity. RNA was denatured (4× RNA denaturing buffer; 65°C, 5 minutes), chilled on ice, neutralized with 20× SSC, adjusted to final volume, and serially diluted. RNA dilutions were applied to prewetted Zeta-Probe membranes (2× SSC) using gentle vacuum. RNA was UV cross-linked (1,200 J/m^2^; Stratalinker), and membranes were blocked and incubated with primary antibodies overnight (4°C) (Cell Signaling Technology, 56593), followed by HRP-conjugated secondary antibodies (Cell Signaling Technology, 7074), ECL development, and imaging (GE HealthCare imager).

### Human pluripotent stem cell culture.

Human embryonic stem cells (H9, WA09; WiCell) and iPSCs were maintained on irradiated mouse embryonic fibroblast feeder layers and passaged using collagenase type IV. Feeder-supported PSCs were cultured in hPSM medium, while feeder-free PSCs were cultured on Matrigel in mTeSR1 medium. Cells were maintained at 37°C under 5% CO_2_.

### shRNA-mediated MYC knockdown.

*MYC* knockdown was performed using lentiviral shRNA constructs targeting human *MYC* (OriGene), with a nontargeting shRNA (shSCR) as control. Lentivirus was produced using standard packaging plasmids, and NPCs were transduced at approximately 80% confluence. After 16–24 hours, virus-containing medium was replaced with fresh NPC medium, and cells were cultured for an additional 48 hours. Knockdown was verified by GFP expression and immunoblotting for c-MYC.

### Extract preparation and immunoblot assays.

Whole-cell lysates were prepared by lysing cells in M-PER mammalian protein extraction reagent (Thermo Fisher Scientific) supplemented with protease and phosphatase inhibitors. Nuclear and cytoplasmic fractions were prepared using a standard sequential extraction protocol. Protein concentrations were measured by Bradford assay. Western blotting was performed using standard procedures with antibodies against c-MYC (Cell Signaling Technology, 9402 and 5605), Neurogenin-2 (Cell Signaling Technology, 13144), and TBP as a nuclear loading control (Cell Signaling Technology, 44059), followed by HRP-conjugated secondary antibodies and chemiluminescent detection.

### Targeted metabolomics.

Metabolites were extracted using cold 80% methanol following saline washes. Tissue samples were homogenized in cold 80% methanol. Extracts were clarified by centrifugation and dried in a SpeedVac. Dried extracts were reconstituted in 80% acetonitrile and analyzed on a Q-Exactive mass spectrometer (Thermo Fisher Scientific) coupled to a Vanquish UHPLC system (Thermo Fisher Scientific) using a ZIC-pHILIC column (MilliporeSigma), as described previously (79–81). Extracted ion chromatograms were integrated for relative quantitation, and metabolite identities were confirmed using authenticated standards and product ion spectra.

### Stable isotope tracing.

For ^13^C-glucose or ^13^C-glutamine tracing, cells were cultured in DMEM/F12 base medium supplemented with 17.5 mM [U-^13^C]glucose or 2.5 mM [U-^13^C]glutamine. Metabolite extraction was performed as described in “Targeted metabolomics.” Natural isotope abundances were corrected using a customized R script, adapted from the AccuCor algorithm (82) available at https://github.com/wencgu/nac (commit ID 15417d4f18ba702afafee4ce6de8d8b0e9243251).

### Immunofluorescence and confocal microscopy.

Cells were seeded onto coverslips and fixed with freshly prepared 4% paraformaldehyde in PBS. Fixed cells were permeabilized, blocked, incubated with primary antibodies followed by Alexa Fluor 488– and Alexa Fluor 555–conjugated secondary antibodies (Invitrogen, A11001 and A21429), mounted with antifade reagent, and cured overnight. Images were acquired on a Zeiss LSM 880 confocal microscope and processed in ImageJ (Fiji). For PAX6 staining (BioLegend, 901301), nuclear signal intensity was quantified in Fiji using DAPI-defined nuclear masks and normalized to DAPI intensity.

### RNA-seq.

Total RNA was extracted using TRIzol reagent (Thermo Fisher Scientific) followed by purification with the RNeasy Mini Kit (Qiagen). RNA-seq libraries were prepared using NEBNext Ultra II Directional RNA Library Prep (New England Biolabs) according to the manufacturer’s instructions. Sequencing reads were aligned to the hg19 reference genome using STAR v.2.5.2b (83), retaining only uniquely mapped reads. Alignment files were processed using BEDtools (v2.29.2) and custom scripts for visualization. Gene-level read counts were obtained using HTSeq (84). Differential expression analysis was performed using DESeq2 (85), with significance defined by an FDR-adjusted *P* value ≤ 0.05.

### ChIP-seq.

ChIP-seq was performed as previously described (86). Crosslinked chromatin was sonicated to approximately 200–500 bp fragments and immunoprecipitated overnight using antibodies against H3K4me2 (Active Motif, 39141), H3K4me3 (Abcam, ab8580), H3K9me3 (Abcam, ab8898), and H3K27me3 (Active Motif, 39155). ChIP DNA was purified, libraries were prepared using the NEBNext Ultra II DNA Library Prep Kit, and sequencing was performed on an Illumina NextSeq 500 platform. Reads were aligned to the human hg19 genome using Bowtie2 (87), and peaks were called using MACS (88).

### scRNA-seq.

Organoids were dissociated into single-cell suspensions using Accutase. Single-cell libraries were generated using the Chromium Next GEM Single Cell 3′ v3.1 kit (10x Genomics) targeting 5,000–10,000 cells per sample and sequenced on an Illumina NovaSeq 6000. Raw FASTQ files were processed using Cell Ranger (v6.1.2) and aligned to the human genome (GRCh38). Downstream analyses were performed in Seurat (v5.0.1). Data were normalized using SCTransform, integrated across samples, and subjected to dimensionality reduction by principal component analysis and UMAP. Cell clusters were identified using the Louvain algorithm, and marker genes were identified using Seurat. Cell-type identities were assigned based on canonical marker expression and reference-based annotation. *MYC* expression differences between corrected and unedited hCSs were assessed in a cell-type–resolved manner using Wilcoxon’s rank-sum tests with FDR correction. Differences in cell-type composition between conditions were quantified and statistically evaluated.

### Statistics.

All statistical analyses were performed using R (v4.3.1). Data are presented as mean ± SEM unless otherwise noted. Comparisons between 2 groups were performed using unpaired 2-tailed Student’s *t* tests, unless otherwise specified. For comparisons involving 3 or more groups, 1- or 2-way ANOVA was used as appropriate, followed by post hoc tests to correct for multiple comparisons. Normality was assessed using the Shapiro-Wilk test, and nonparametric tests were applied when appropriate. Statistical details are provided in the legends. A *P* value < 0.05 was considered statistically significant.

### Study approval.

All human studies were approved by the IRB at UTSW (NCT02650622). Written informed consent was obtained from all participants. Procedures involving animals were approved by the UTSW IACUC.

### Data availability.

All sequencing data have been deposited in the Gene Expression Omnibus under accession numbers GSE295010 (RNA-seq) and GSE295011 (ChIP-seq). Additional raw and processed data are available upon reasonable request. Supplemental material includes 6 figures and 8 tables. Values for all data points in graphs are provided in the Supporting Data Values file.

## Author contributions

WG designed the study, conducted experiments, analyzed results, and wrote the manuscript. XW contributed to ChIP-seq experiments. AS, BF, ZW, JS, LGZ, FC, and TPM contributed to metabolomics experiments. LC, YX, YZ, and AKW assisted with sequencing data analysis. AT, JF, AR, HT, and BA provided support with cell culture experiments. HZ and IB carried out Ca^2+^ imaging experiments and data analysis. SS contributed to mouse experiments. SKM provided guidance on the design and interpretation of epigenetic analyses. RJD provided funding, designed the study, analyzed results, and wrote the manuscript.

## Conflict of interest

SKM receives support from Servier Pharmaceuticals and is a cofounder of Gliomet. RJD is a founder and advisor at Atavistik Bioscience and an advisor at Vida Ventures, Agios Pharmaceuticals, Illumina, and Faeth Therapeutics.

## Funding support

This work is the result of NIH funding, in whole or in part, and is subject to the NIH Public Access Policy. Through acceptance of this federal funding, the NIH has been given a right to make the work publicly available in PubMed Central.

- Howard Hughes Medical Institute (HHMI) Investigator Program (RJD).
- NIH: R35CA220449 (RJD); R01CA289260, R01CA258586, P50CA165962, and U19CA264504 (SKM); R01CA200653 (SS); R01CA285336 (LC); K99CA277576 (YX).
- Cancer Prevention Research Institute of Texas: RP220337 (RJD); RP240494 (LGZ, TPM, and the Children’s Medical Center Research Institute Metabolomics Facility); RR190034, RP230344, and RP2400489 (SKM).
- Moody Foundation (Robert L. Moody, Sr. Faculty Scholar Award) (RJD).
- Eugene McDermott Endowment for the Study of Human Growth and Development (RJD).
- Human Frontier Science Program: LT0018/2022-L (YX).

## Supplementary Material

Supplemental data

Unedited blot and gel images

Supplemental table 1

Supplemental table 2

Supplemental table 3

Supplemental table 4

Supplemental table 5

Supplemental table 6

Supplemental table 7

Supplemental table 8

Supporting data values

## Version 1 03/17/2026

In-Press Preview

## Version 2 05/01/2026

Electronic publication
