# Supplementary material for: l-2-Hydroxyglutarate impairs neuronal differentiation through epigenetic activation of MYC expression
Source: J Clin Invest. 2026 Mar 17;136(9):e197010. doi: 10.1172/JCI197010 (PMC13132370; doi:10.1172/JCI197010)
Supplement: Supplemental data [file jci-136-197010-s253.pdf]

L-2-hydroxyglutarate impairs neuronal differentiation through epigenetic activation of *MYC* expression

Wen Gu[1], Xun Wang[1,2], Ashley Solmonson[1], Ling Cai[1,3], Yi Xiao[1], Alpaslan Tasdogan[1,4], Jordan Franklin[1], Yuannyu Zhang[1], Hua Zhang[5,6], Aundrea K. Westfall[1], Ashley Rowe[1], Hetali Trivedi[1], Brandon Faubert[1], Zheng Wu[1], Jessica Sudderth[1], Lauren G. Zacharias[1], Bushra Afroze[7], Ilya Bezprozvanny[5,8], Sunil Sudarshan[9], Feng Cai[1], Samuel K. McBrayer[1,10], Thomas P. Mathews[1], Ralph J. DeBerardinis[1,11,12]*

[1]Children's Medical Center Research Institute, University of Texas Southwestern Medical Center, Dallas, TX, USA

[2]Current affiliation: State Key Laboratory of Common Mechanism Research for Major Diseases, Suzhou Institute of Systems Medicine, Chinese Academy of Medical Sciences & Peking Union Medical College, Suzhou, China

[3]Quantitative Biomedical Research Center, Peter O'Donnell Jr. School of Public Health, University of Texas Southwestern Medical Center, Dallas, TX, USA

[4]Current affiliation: Department of Dermatology, University Hospital Essen and German Cancer Consortium, Partner Site Essen, Essen, Germany

[5]Department of Physiology, University of Texas Southwestern Medical Center, Dallas, TX, USA

[6]Department of Pathology, University of Texas Southwestern Medical Center, Dallas, TX, USA

[7]Department of Paediatrics & Child Health, Aga Khan University, Stadium Road, Karachi, Pakistan

[8]Current affiliation: Peter the Great St Petersburg Polytechnical University, St Petersburg, Russian Federation

[9]Department of Urology, University of Alabama at Birmingham, Birmingham, Alabama, USA

[10]Peter O'Donnell Jr. Brain Institute, University of Texas Southwestern Medical Center, Dallas, TX, USA

[11]Eugene McDermott Center for Human Growth and Development, University of Texas Southwestern Medical Center, Dallas, TX, USA

[12]Howard Hughes Medical Institute, University of Texas Southwestern Medical Center, Dallas, TX, USA

Address correspondence to: Ralph J. DeBerardinis, Eugene McDermott Center for Human Growth and Development, University of Texas Southwestern Medical Center, 5323 Harry Hines Blvd., Dallas, Texas 75390, USA. Phone: 214.648.2587; Email: ralph.deberardinis@utsouthwestern.edu.

Conflict of interest: Samuel K. McBrayer receives support from Servier Pharmaceuticals and is a co-founder of Gliomet. Ralph J. DeBerardinis is a founder and advisor at Atavistik Bioscience, and an advisor at Vida Ventures, Agios Pharmaceuticals, Illumina, and Faeth Therapeutics.

SUPPLEMENTAL FIGURES

Supplemental Figure 1

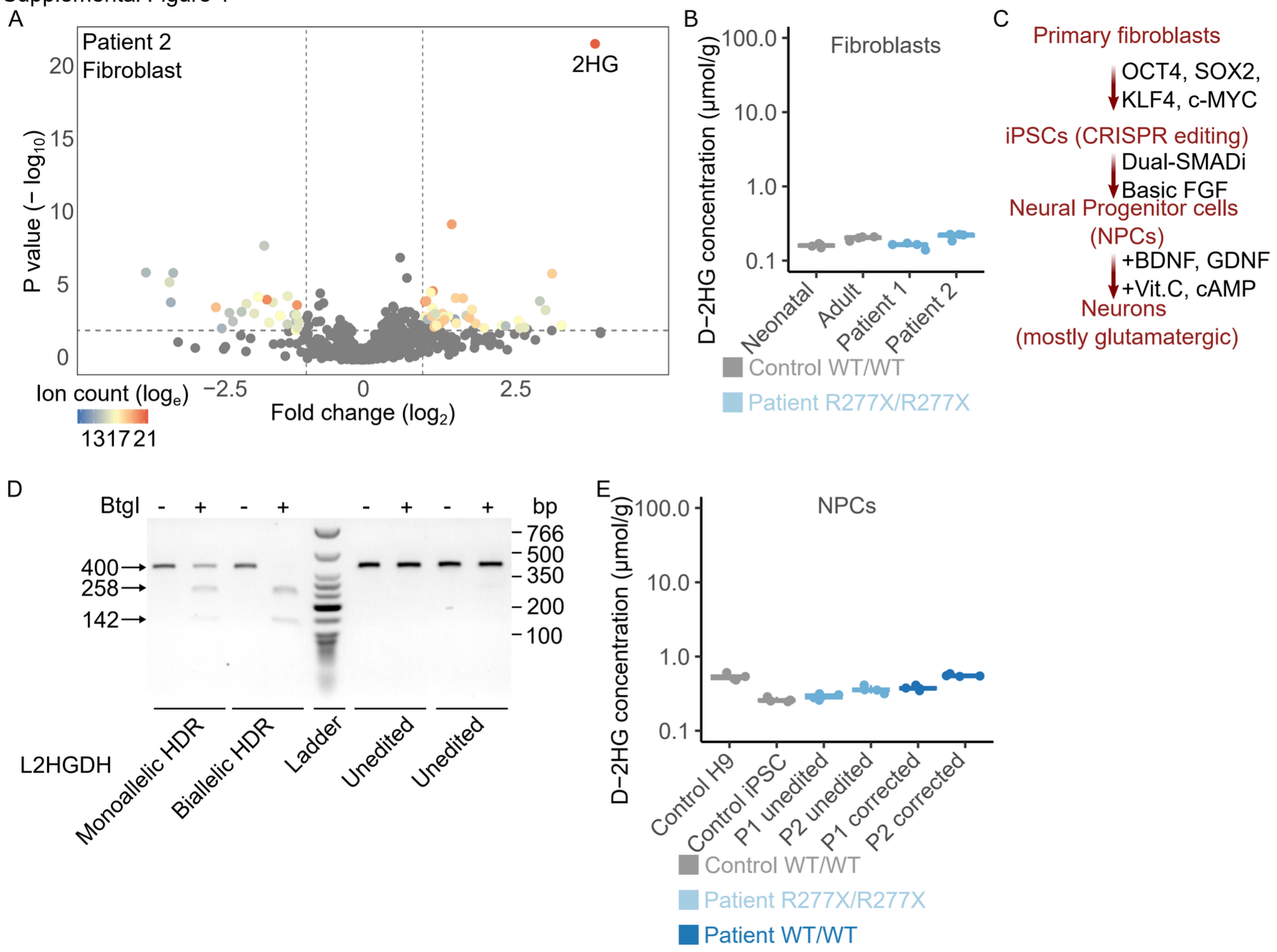

Supplemental Figure 1. An isogenic, patient-derived iPSC system to study L2HGDH deficiency.

(A) Volcano plot of fibroblast metabolites comparing Patient 2 to 29 unrelated lines (sibling excluded), each profiled in quadruplicate. A mixed-effects model was used as in Figure 1C, with cell line as the random effect.

(B) D-2HG concentrations in neonatal control fibroblasts, adult control fibroblasts, and L2HGA patient fibroblasts.

(C) Schematic of neuronal differentiation using patient-derived induced pluripotent stem cells (iPSCs), starting from patient fibroblasts and progressing through NPCs to mature neurons (predominantly glutamatergic).

(D) Restriction fragment length polymorphism (RFLP) analysis of iPSC clones following CRISPR/Cas9 genome editing, showing the digestion pattern with *Btg*I to differentiate between unedited, monoallelic HDR, and biallelic HDR clones.

(E) D-2HG concentrations in neural progenitor cells (NPCs) derived from various hPSC lines, including control H9 NPCs, control iPSC-derived NPCs, Patient 1 unedited NPCs, Patient 2 unedited NPCs, Patient 1 corrected NPCs, and Patient 2 corrected NPCs.

For (B) and (E), data are shown as box plots with jittered individual data points. Boxes indicate the interquartile range (25th–75th percentile), with the horizontal line marking the median. Whiskers extend to the minimum and maximum values within 1.5× the interquartile range. Statistical significance was assessed using one-way ANOVA followed by Tukey's HSD test (n = 4 per group).

Supplemental Figure 2

A

Top 30 GSEA normalized enrichment scores (NES) in corrected vs. unedited neurons

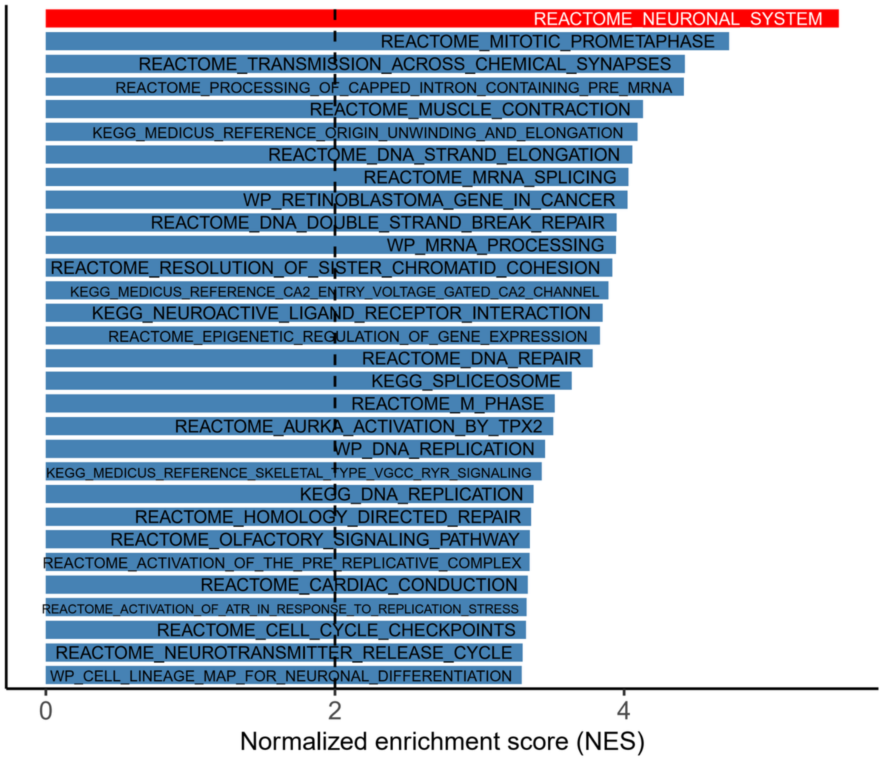

B

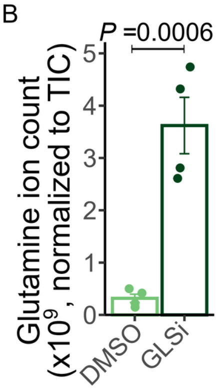

C

Replicate	Glutamine consumption (nmol/plate)	Intracellular L-2HG (nmol/plate)	Extracellular L-2HG (nmol/plate)	Labeled L-2HG, intracellular (%)	Labeled L-2HG, extracellular (%)	Total labeled L-2HG (nmol/plate)	Glutamine→L-2HG in 4 h (%)
1	259.11	2.82	2.78	25.56%	22.97%	1.36	0.52%
2	376.21	3.61	4.08	25.44%	22.76%	1.85	0.49%
3	342.44	2.80	2.50	25.99%	23.57%	1.32	0.38%
Mean ± SEM	325.92 ± 34.80	3.08 ± 0.27	3.12 ± 0.49	25.66% ± 0.17%	23.10% ± 0.24%	1.51 ± 0.17	0.46% ± 0.04%

D

Replicate	Glutamate secreted (nmol/plate)	Glutamine consumption (nmol/plate)	Glutamine→glutamate secreted in 4 h (%)
1	52.72	259.11	20.34%
2	57.23	376.21	15.21%
3	51.37	342.44	15.00%
Mean ± SEM	53.77 ± 1.77	325.92 ± 34.80	16.85% ± 1.75%

E

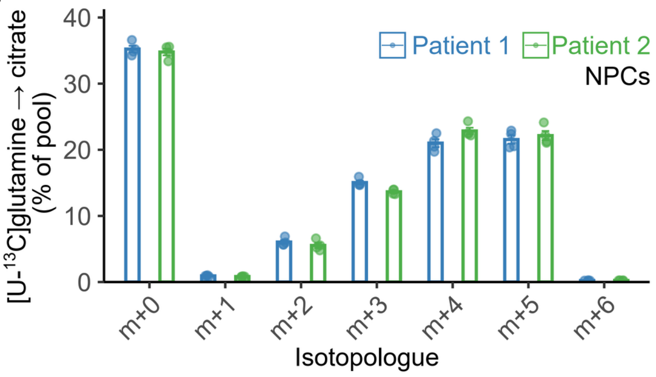

Supplemental Figure 2. Neuronal pathway enrichment in neurons and quantitative routing of glutamine carbon to L-2HG, glutamate, and citrate in NPCs.

(A) Top 30 enriched gene sets from GSEA comparing corrected neurons vs unedited neurons (MSigDB c2.cp). Bars show normalized enrichment scores (NES); dashed line marks NES = 2.0.

(B) Intracellular glutamine abundance after 4 h treatment with DMSO or CB-839 (GLSi) at the dose used in functional assays; LC–MS ion counts were normalized to total ion current (TIC). Data were non-normally distributed (Shapiro–Wilk), so statistical significance was assessed by Mann–Whitney/Wilcoxon rank-sum test (n = 4 per group; two-sided; exact P reported in panel).

(C) Quantification of [U-^{13}C]glutamine consumption and L-2HG production over 4 h in unedited NPCs. The table lists glutamine consumed per plate and intra-/extracellular L-2HG (nmol/plate), % labeled fractions, and total labeled L-2HG; mean ± SEM shown.

(D) Glutamate secretion over 4 h in the same experiments as (B). The table reports glutamate secreted (nmol/plate), glutamine consumption, and the % of consumed glutamine released as glutamate; mean ± SEM shown.

(E) [U-^{13}C]glutamine→citrate isotopologue distribution (m+0–m+6) in unedited NPCs from Patient 1 and Patient 2, indicating contributions from oxidative TCA cycle metabolism (m+4) and reductive carboxylation (m+5).

Supplemental Figure 3

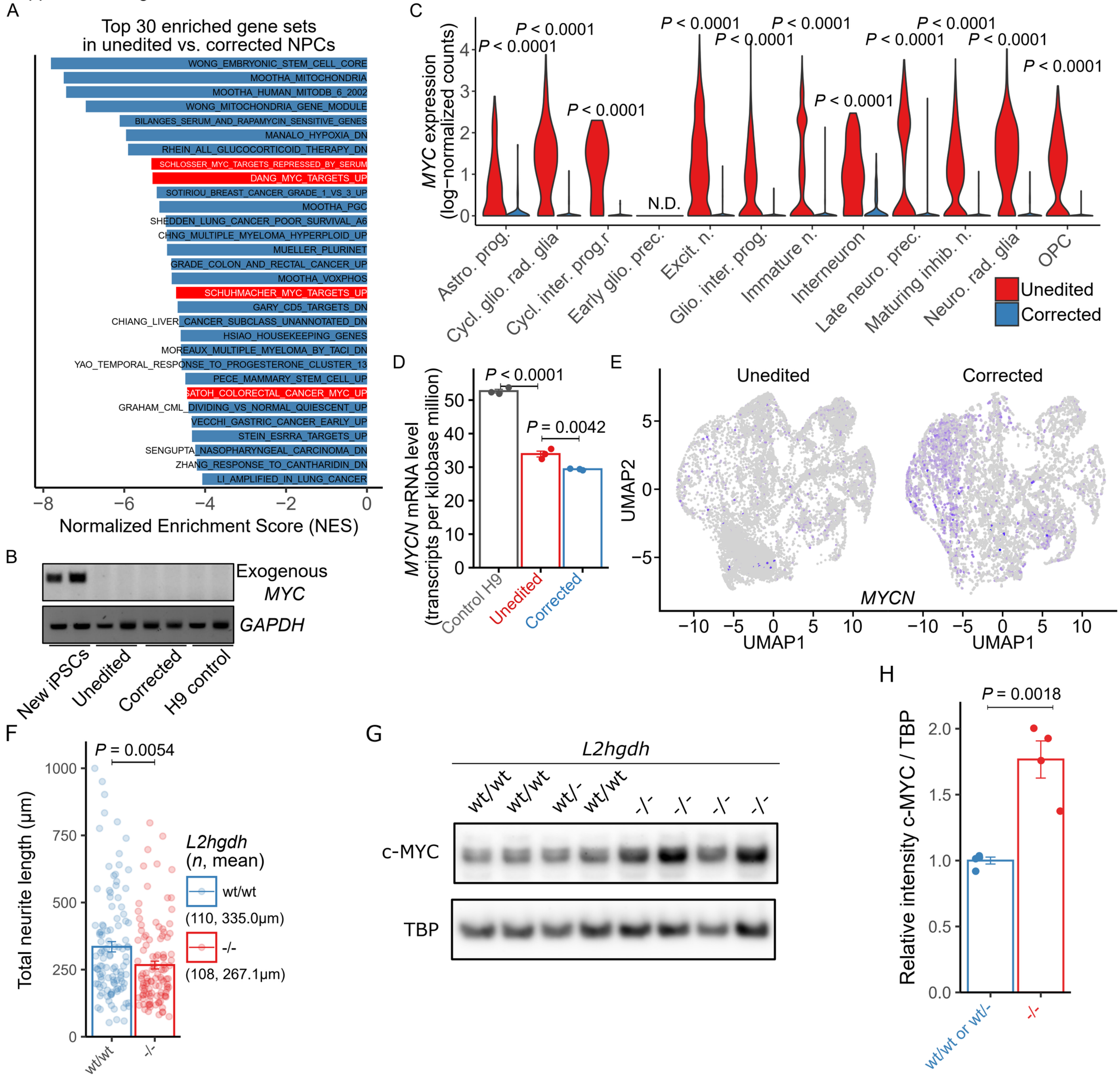

Supplemental Figure 3. Increased *MYC* expression in L2HGDH-deficient NPCs.

(A) Top 30 enriched gene sets from GSEA comparing unedited vs corrected NPCs (MSigDB c2.cgp). Bars show normalized enrichment score (NES); *MYC*-related sets are highlighted; dashed line marks NES = 2.0.

(B) RT-PCR detecting exogenous (Sendai-derived) *MYC* using Sendai-specific primers. The product is present in newly reprogrammed iPSCs and absent in unedited and corrected NPCs and H9 control; GAPDH control shown.

(C) Single-cell RNA-seq violin plots showing *MYC* expression (log-normalized counts) across day-45 cortical spheroid lineages. N.D., not detected.

(D) Quantification of *MYCN* mRNA in control H9 NPCs, unedited Patient 1 NPCs, and corrected Patient 1 NPCs. mRNA levels were obtained from RNA sequencing of biological triplicates and expressed as transcripts per kilobase million (TPM).

(E) UMAP feature plots of *MYCN* expression in day-45 cortical spheroids from unedited vs corrected cultures.

(F) Quantification of total neurite lengths in wild-type (*wt/wt*) and *L2hgdh*$^{-/-}$ neurons derived from mouse NPCs.

(G) Immunoblot analysis of nuclear c-MYC in wild-type (*wt/wt*) and *L2hgdh*$^{-/-}$ NPCs derived from E15.5 mouse embryos. TBP was used as a loading control for nuclear lysates.

(H) Quantification of relative c-MYC levels, normalized to TBP, as described in (G).

Error bars represent ± 1 SEM of biological replicates. For (C), expression values reflect normalized, log-transformed transcript counts. For each cell type, statistical significance was assessed using a two-sided Wilcoxon rank-sum test. *P* values were adjusted for multiple comparisons using the Benjamini-Hochberg method. For (D), statistical significance was assessed on log-transformed TPM values using one-way ANOVA followed by Tukey’s HSD test. Error bars represent ±1 SEM of three biological replicates. For (F) and (H), statistical significance was determined using Student’s t-test. For (F), sample sizes and mean neurite lengths are indicated in the figure; for (H), n = 4.

Supplemental Figure 4

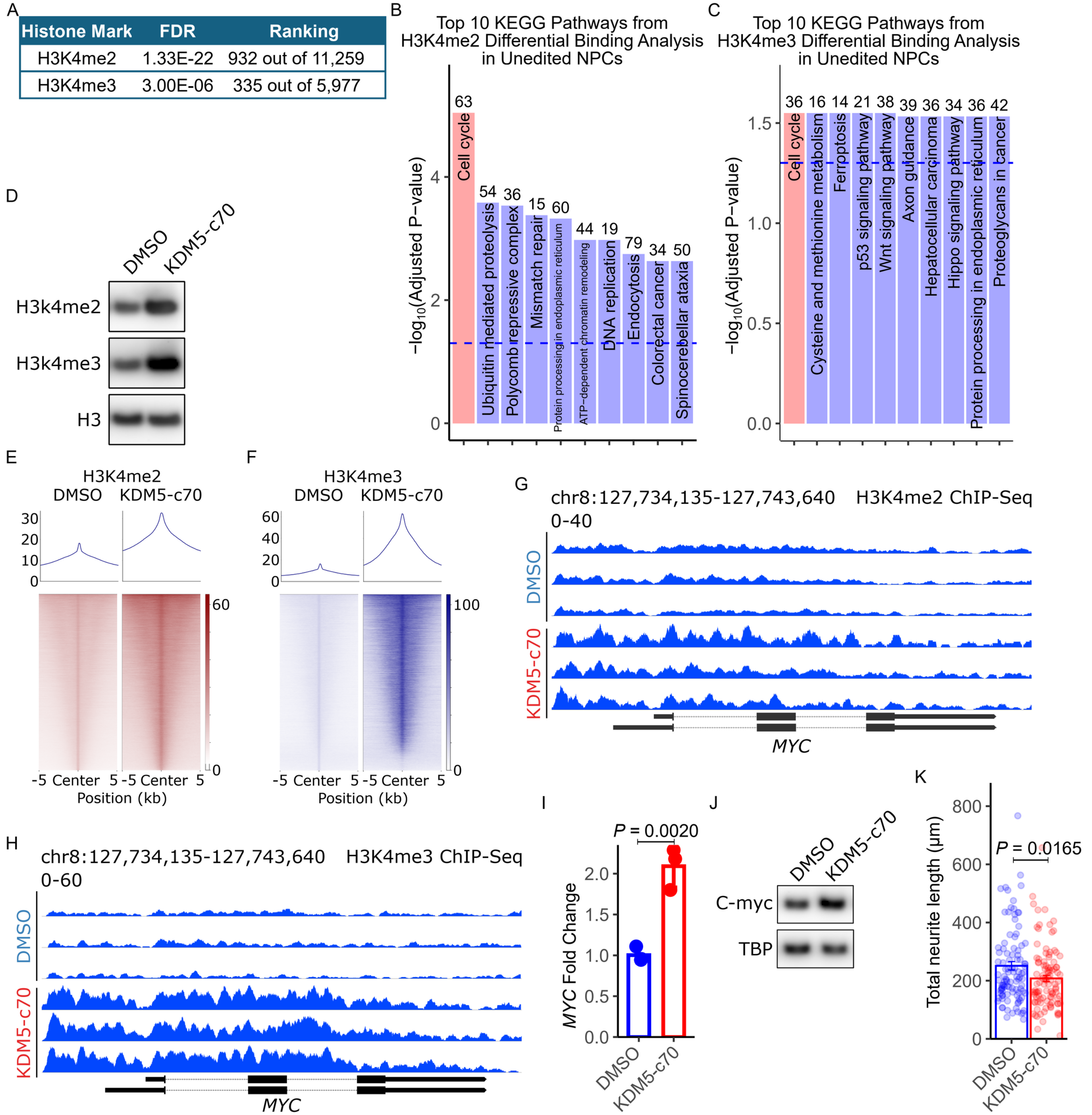

Histone Mark	FDR	Ranking
H3K4me2	1.33E-22	932 out of 11,259
H3K4me3	3.00E-06	335 out of 5,977

Supplemental Figure 4. Increased activating histone methylation marks H3K4me2 and H3K4me3 at the *MYC* locus in L2HGDH-deficient NPCs.

(A) Statistical significance of differential binding analysis for H3K4me2 and H3K4me3 markers, comparing unedited vs. corrected Patient 1 NPCs. The adjusted false discovery rate (FDR) ranking of each histone mark is shown. The third column shows the ranking of the *MYC* locus among all differential loci.

(B) and (C) Top 10 KEGG pathways identified from differential binding analysis of H3K4me2 (B) and H3K4me3 (C) in unedited vs. corrected Patient 1 NPCs, based on ChIP-seq data. The Y-axis represents the $-\log_{10}$ (*P*-value) of pathway enrichment, while the columns indicate the top 10 enriched pathways. Numbers above the bars indicate the number of genes associated with each pathway. The blue dashed line marks the statistical significance threshold at $P = 0.05$.

(D) Immunoblot analysis of activating histone marks H3K4me2 and H3K4me3 in corrected Patient 1 NPCs treated with DMSO or the KDM5 inhibitor C70 (25 μM) for 14 days. Histone H3 was used as a loading control for histone extraction.

(E) and (F) ChIP–Seq profiles of H3K4me2 (E) and H3K4me3 (F) in corrected Patient 1 NPCs treated with DMSO or the KDM5 inhibitor C70 (25 μM) for 14 days. ChIP–Seq signals were plotted over center peaks (± 5 kb from peak center) identified in DMSO-treated NPCs. Sites were sorted by the ChIP–Seq signal intensity from DMSO-treated NPCs.

(G) and (H) Representative ChIP-seq tracks showing H3K4me2 (G) and H3K4me3 (H) enrichment at the *MYC* locus in corrected Patient 1 NPCs treated with DMSO or the KDM5 inhibitor C70 (25 μM) for 14 days.

(I) qRT-PCR analysis of *MYC* mRNA levels (normalized to 36B4) in corrected Patient 1 NPCs treated with DMSO or KDM5 inhibitor C70 (25 μM) for 14 days. Statistical analysis was performed to compare qPCR fold change values between the DMSO and C70 groups. First, data normality was assessed using the Shapiro-Wilk test, confirming that both groups followed a normal distribution ($P > 0.05$). Based on this, an independent t-test was applied, yielding a statistically significant result ($P = 0.0020$). $n = 3$ biological replicates.

(J) Immunoblot analysis of nuclear c-MYC levels in corrected Patient 1 NPCs treated with DMSO or the KDM5 inhibitor C70 (25 μM) for 14 days. TBP was used as a loading control for nuclear lysates.

(K) Quantification of total neurite lengths in corrected Patient 1 NPCs treated as described in (J). Neurite lengths were measured using the SNT plugin in ImageJ from immunofluorescent images of fixed cells. Data are presented as box plots with jittered individual data points. Boxes represent the interquartile range (25th–75th percentile), the horizontal line indicates the median, and whiskers extend to values

within 1.5× the interquartile range. Statistical significance was determined by Student’s t-test (n = 105 per group).

Supplemental Figure 5

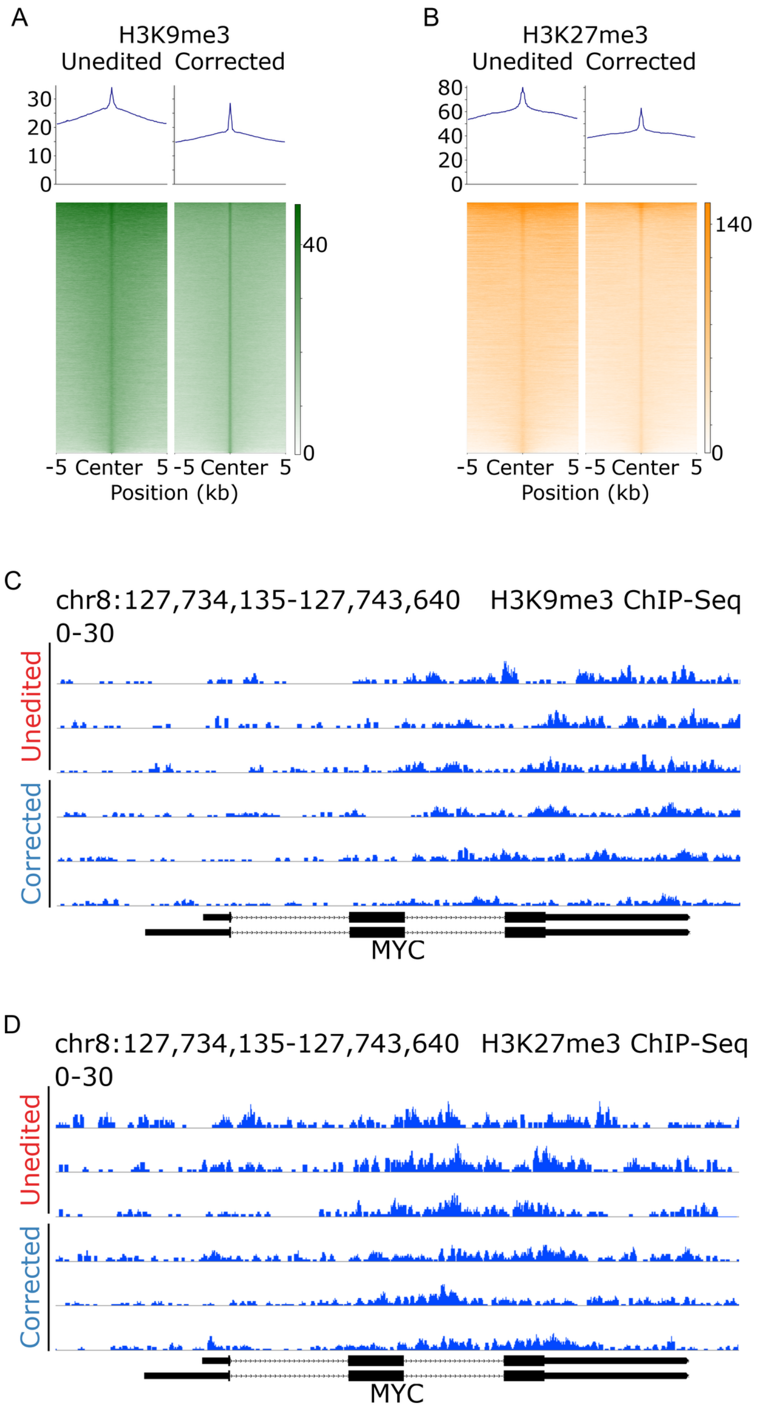

Supplemental Figure 5. Genome-wide gains of repressive histone methylation in L2HGDH-deficient NPCs without enrichment at the *MYC* locus.

(A) and (B) ChIP–Seq profiles of H3K9me3 (A) and H3K27me3 (B) in unedited versus corrected NPCs. Signals are plotted over peak centers (±5 kb from peak center) identified in corrected NPCs. Heatmaps are sorted by ChIP–Seq signal intensity in corrected NPCs. Metaprofiles (top) and heatmaps (bottom) show higher genome-wide signal in unedited cells.

(C) and (D) Representative genome browser tracks showing H3K9me3 (C) and H3K27me3 (D) across the *MYC* locus (chr8:127,734,135–127,743,640). In contrast to the global increases in (A–B), repressive marks are not elevated at the *MYC* locus in unedited cells. Track scales as shown in the panels.

Supplemental Figure 6

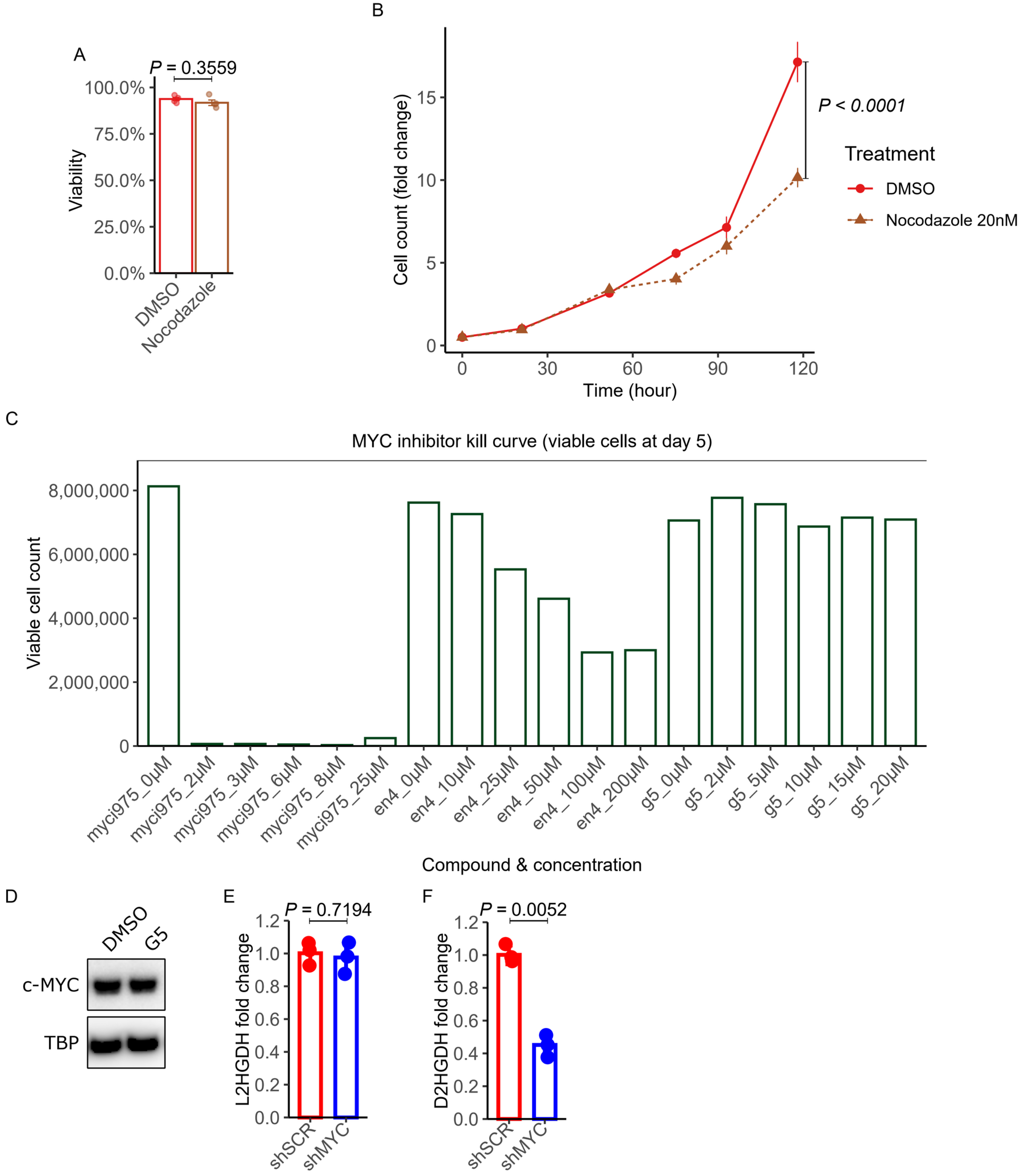

Supplemental Figure 6. c-MYC depletion restores neuronal differentiation in L2HGDH-deficient NPCs.

(A) Cell viability analysis of unedited Patient 1 NPCs treated with DMSO or 20 μM nocodazole for 7 days. Cell viability was measured as a percentage of total viable cells in each condition. Error bars represent ± 1 SEM of three biological replicates. Statistical significance was determined using Student's t-test.

(B) Growth curves of unedited Patient 1 NPCs treated with DMSO or 20 μM nocodazole. Error bars represent ±1 SEM of biological replicates. Differences in cell growth over time were assessed using a two-way ANOVA on fold-change data, with treatment and time point as independent factors. Three biological replicates were included for each condition at each time point, with an assumption of independent sampling across groups

(C) c-MYC inhibitor kill curve (viable cell counts at day 5) across compounds and doses: MYCi975, EN4, and 10074-G5 (G5).

(D) Immunoblot of nuclear c-MYC in NPCs treated with DMSO or 10074-G5; TBP is the nuclear loading control.

(E) qRT-PCR analysis of *L2HGDH* mRNA in unedited NPCs transduced with shSCR or shMYC. Values were normalized to 36B4 and expressed as fold change relative to shSCR. Data normality was assessed with the Shapiro–Wilk test (both groups $P > 0.05$), justifying a parametric comparison. An independent two-tailed t-test showed no significant difference ($P = 0.7194$). n = 3 biological replicates; bars show mean with individual points.

(F) qRT-PCR analysis of *D2HGDH* mRNA in the same samples as in (E), normalized to 36B4 and expressed as fold change relative to shSCR. Shapiro–Wilk confirmed normality for both groups ($P > 0.05$). An independent two-tailed t-test revealed a significant reduction with shMYC ($P = 0.0052$). n = 3 biological replicates; bars show mean with individual points.
