# Supplementary material for: l-2-Hydroxyglutarate impairs neuronal differentiation through epigenetic activation of MYC expression
Source: J Clin Invest. 2026 Mar 17;136(9):e197010. doi: 10.1172/JCI197010 (PMC13132370; doi:10.1172/JCI197010)
Supplement: Unedited blot and gel images [file jci-136-197010-s254.pdf]

Blot and gel images

Full unedited blots for Figure 4C

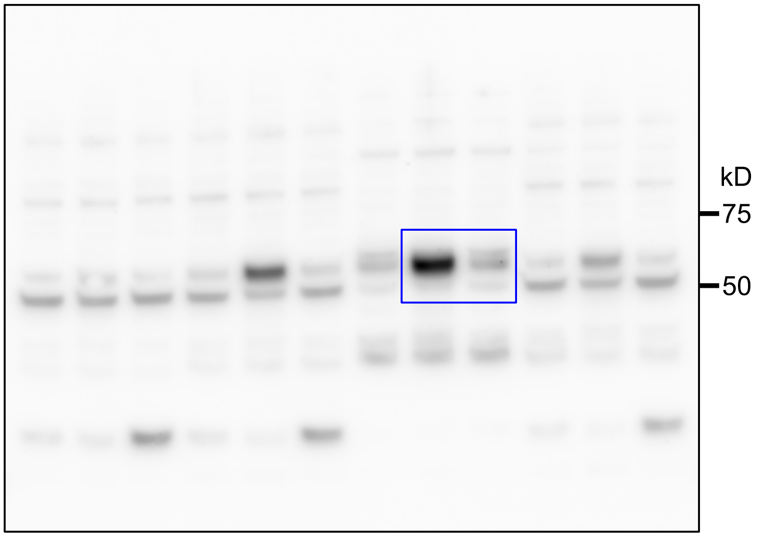

c-MYC

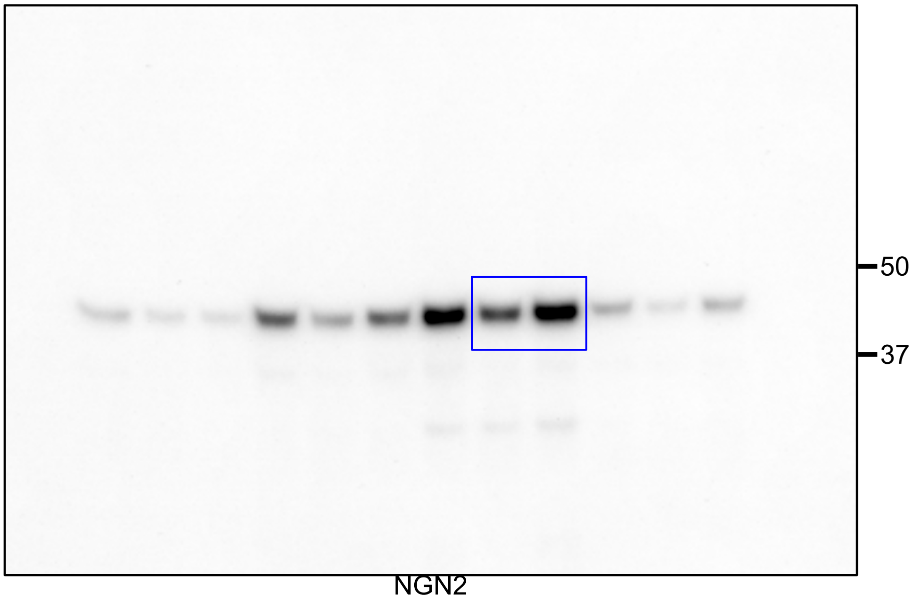

NGN2

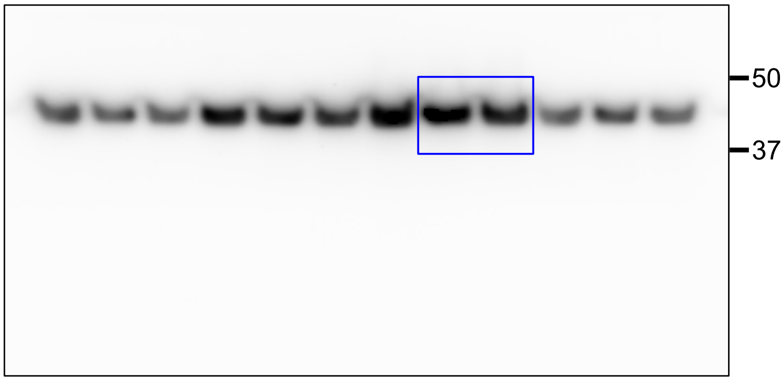

TBP

Blot and gel images

Full unedited blots for Figure 4F

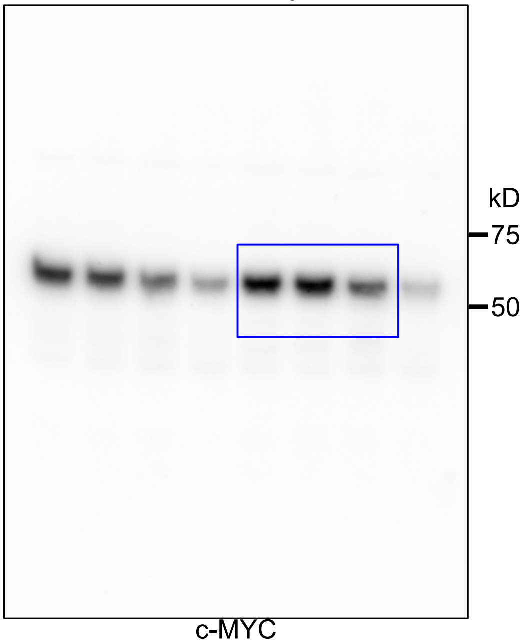

c-MYC

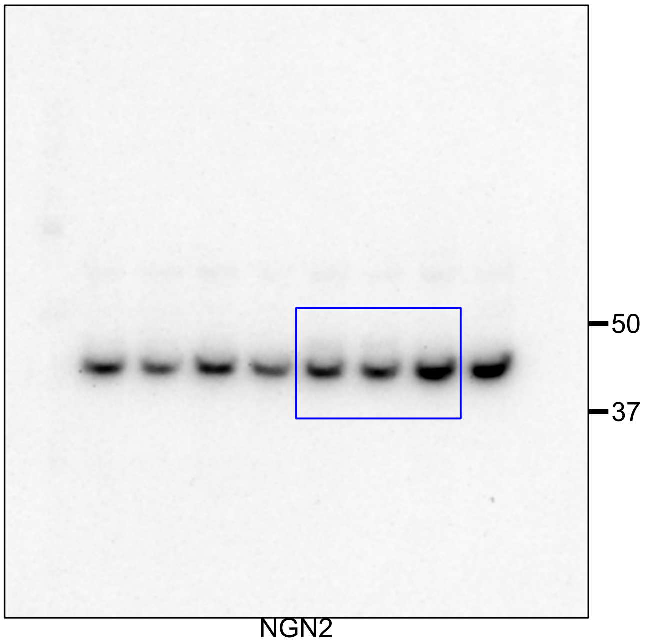

NGN2

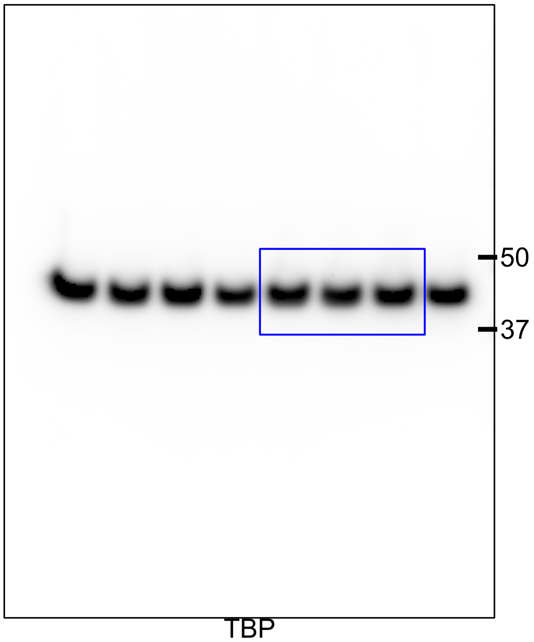

TBP

Blot and gel images

Full unedited blots for Figure 5C

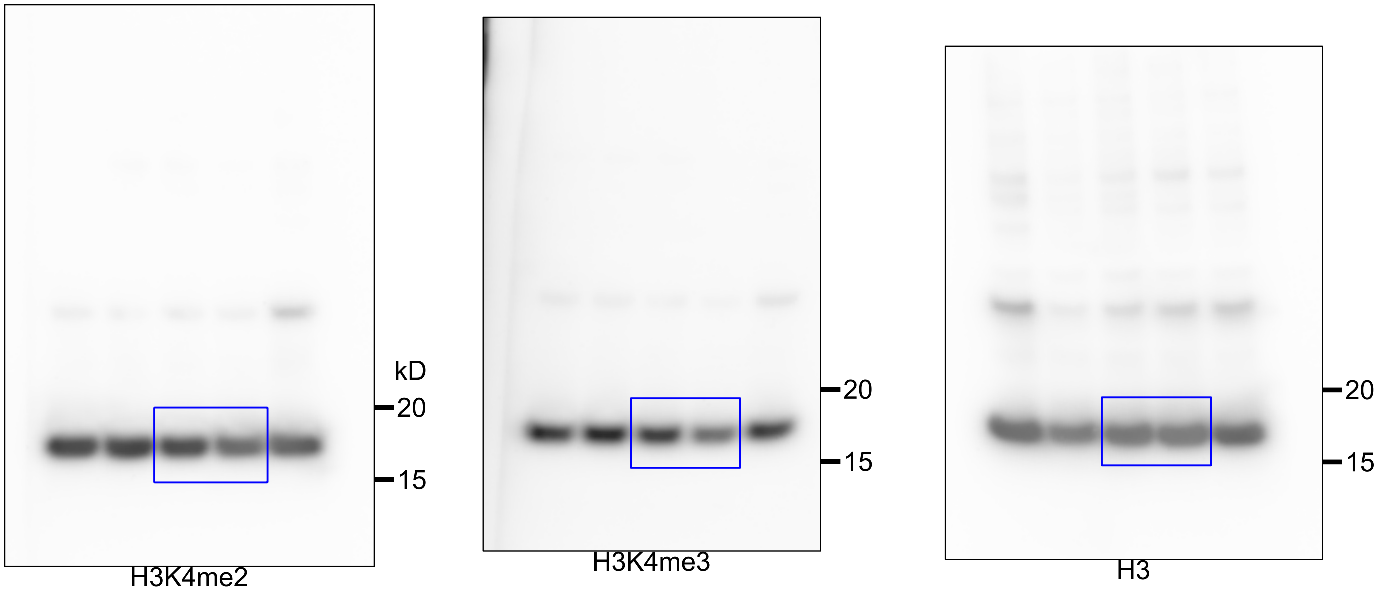

Blot and gel images

Full unedited blots for Figure 6A

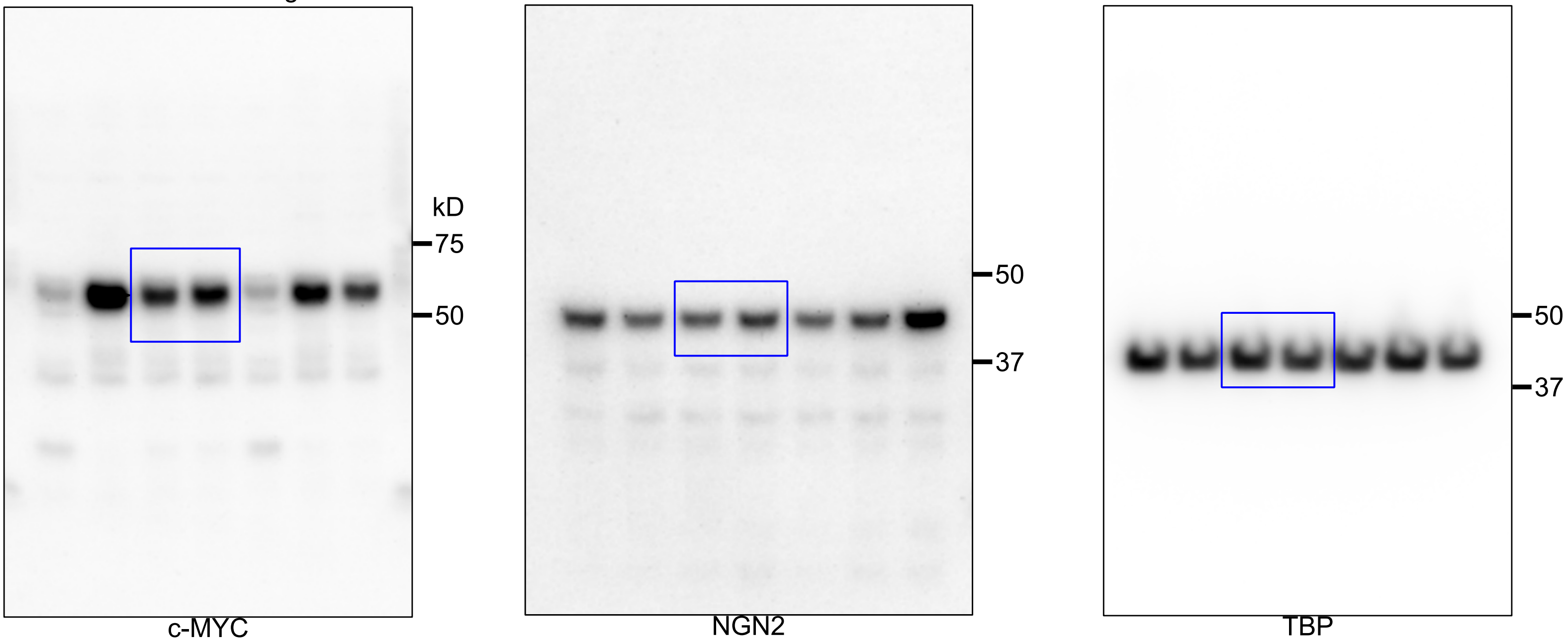

Blot and gel images

Full unedited blots for Figure 6C

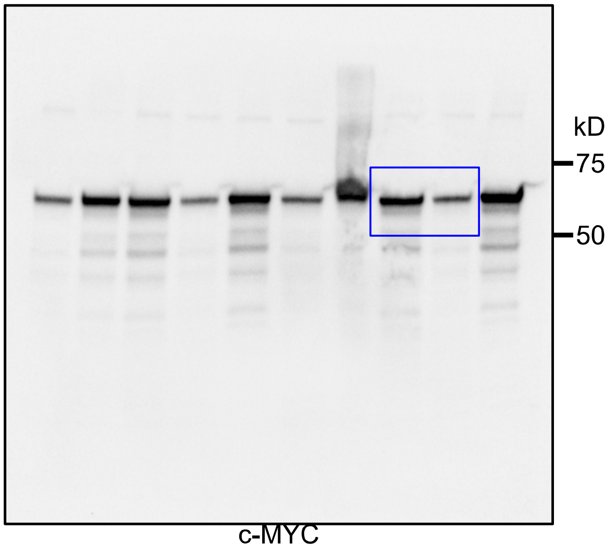

c-MYC

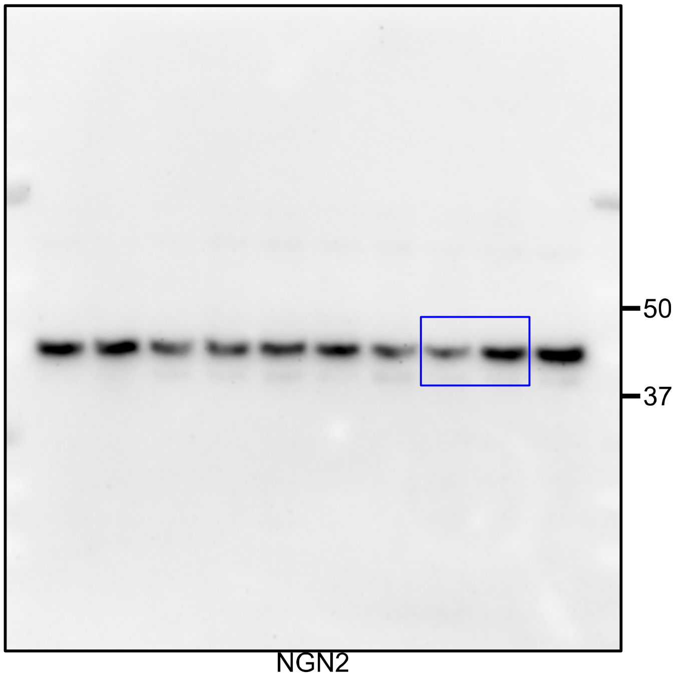

NGN2

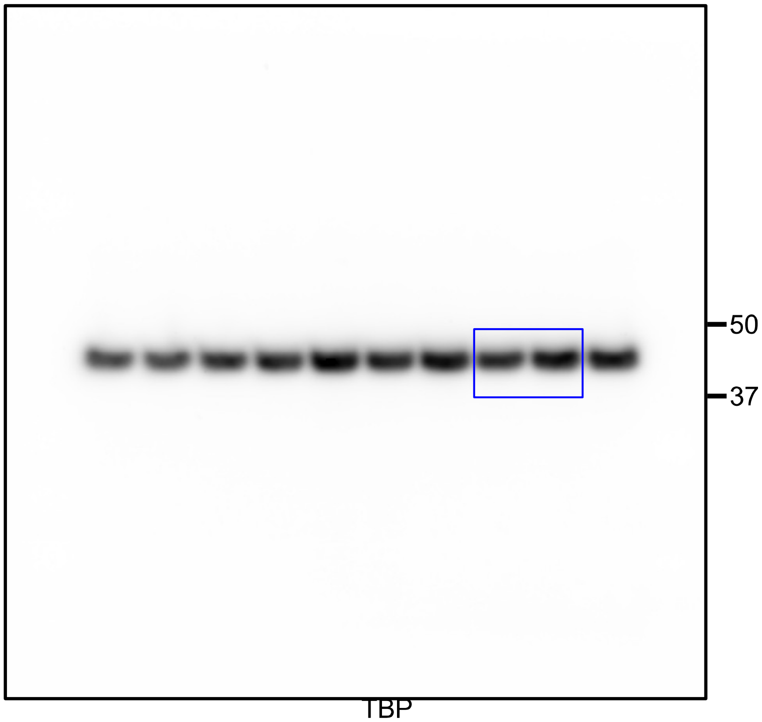

TBP

Blot and gel images

Full unedited blots for Figure 6F

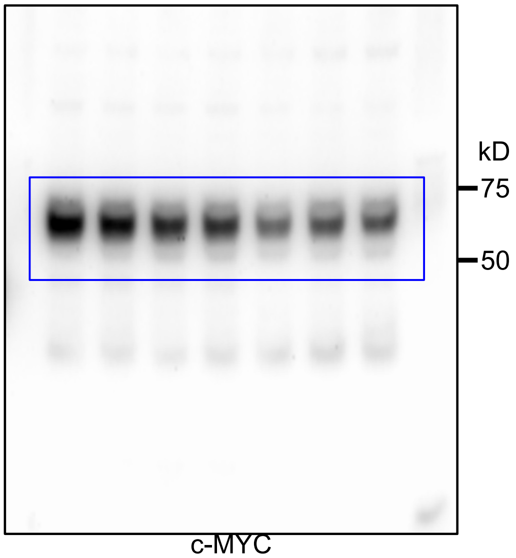

c-MYC

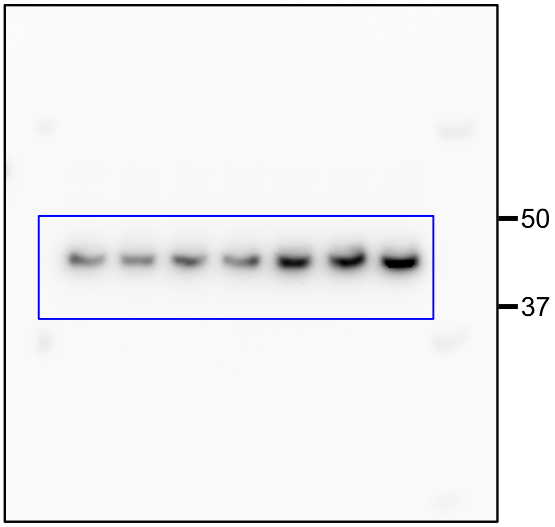

NGN2

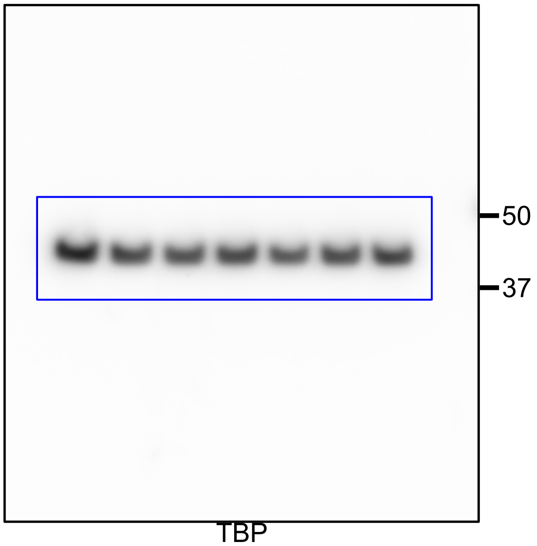

TBP

Blot and gel images

Full unedited gels for Supplemental Figure 3B

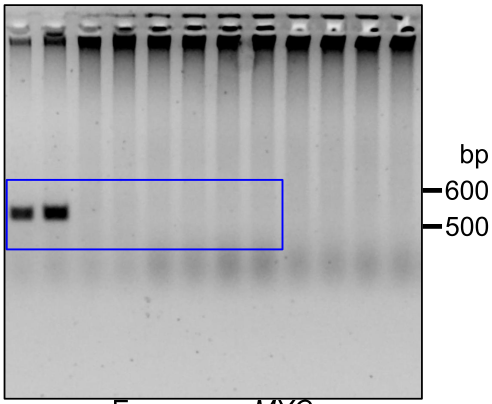

Exogenous *MYC*

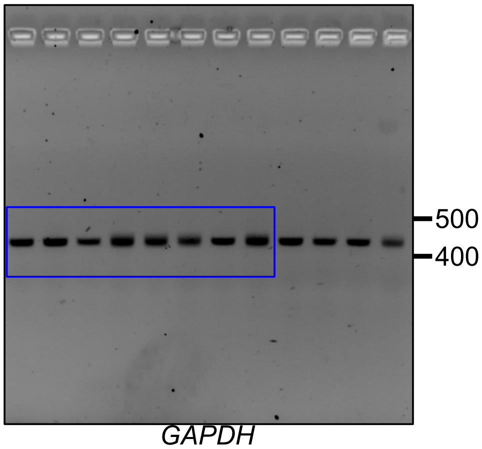

GAPDH

Blot and gel images

Full unedited blots for Supplemental Figure 3G

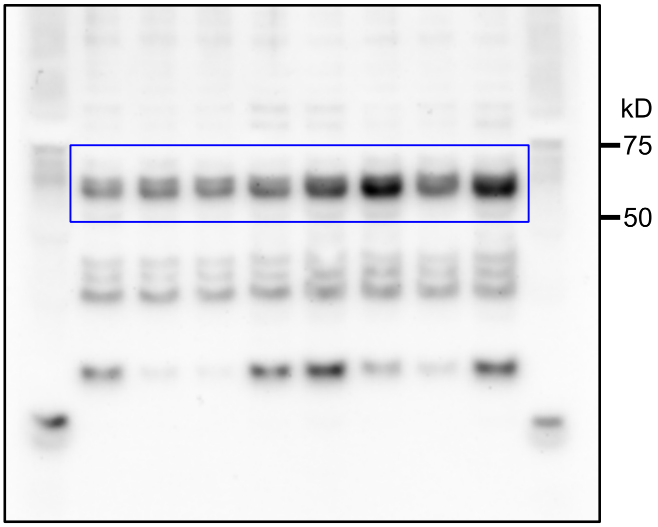

c-MYC

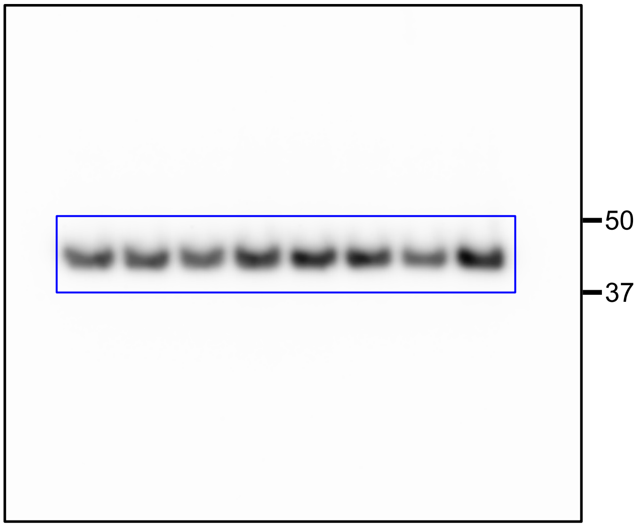

TBP

Blot and gel images

Full unedited blots for Supplemental Figure 4D

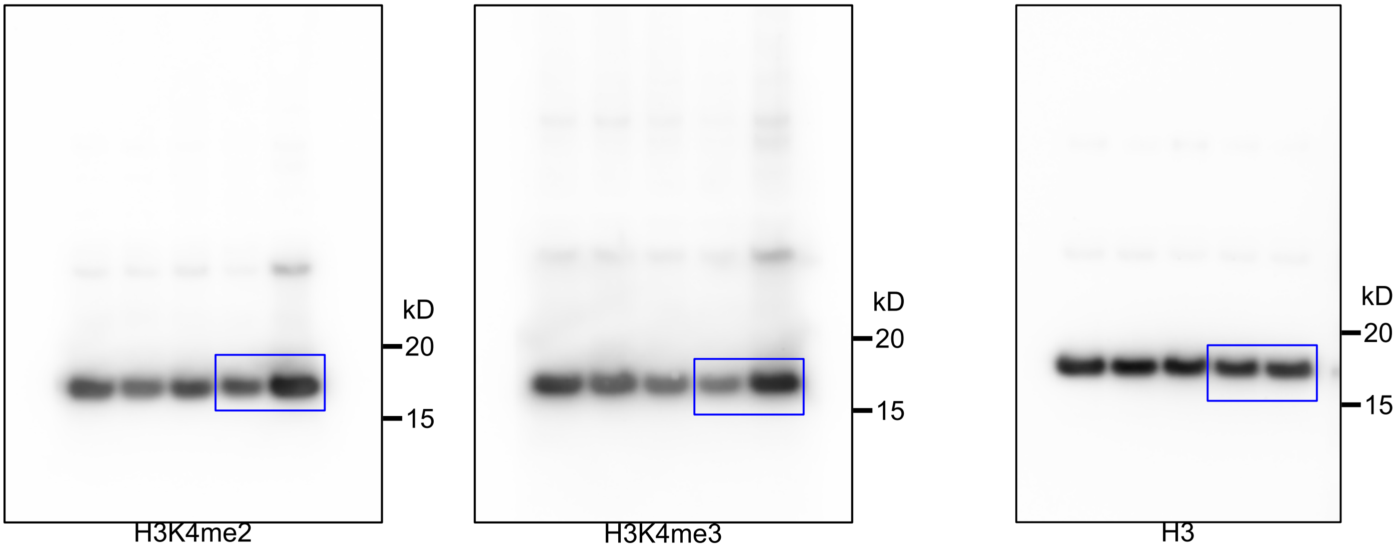

Blot and gel images

Full unedited blots for
Supplemental Figure 4J

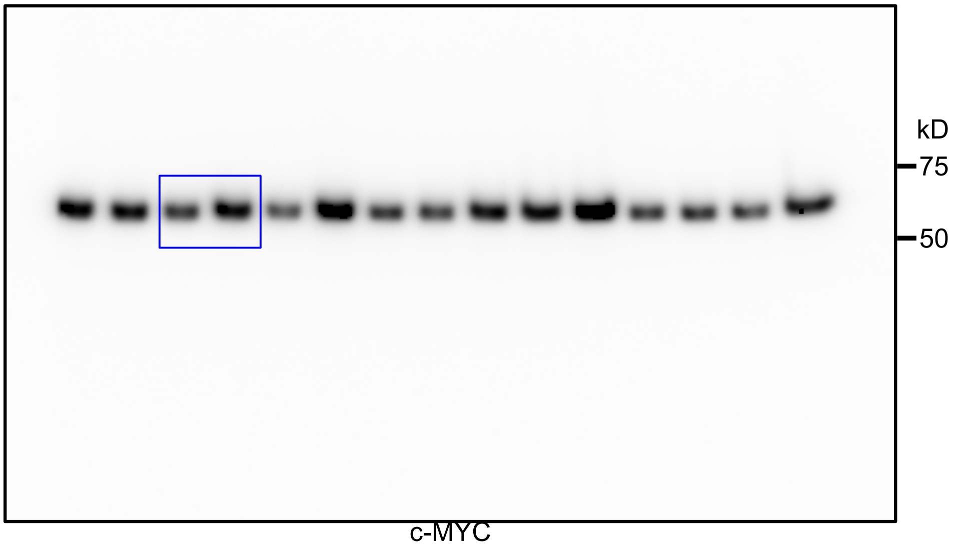

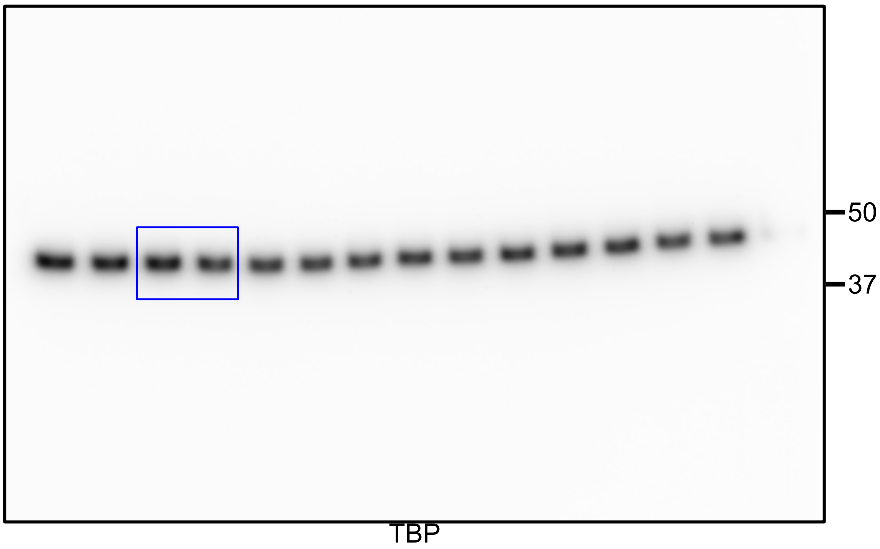

Blot and gel images

Full unedited blots for
Supplemental Figure 6D

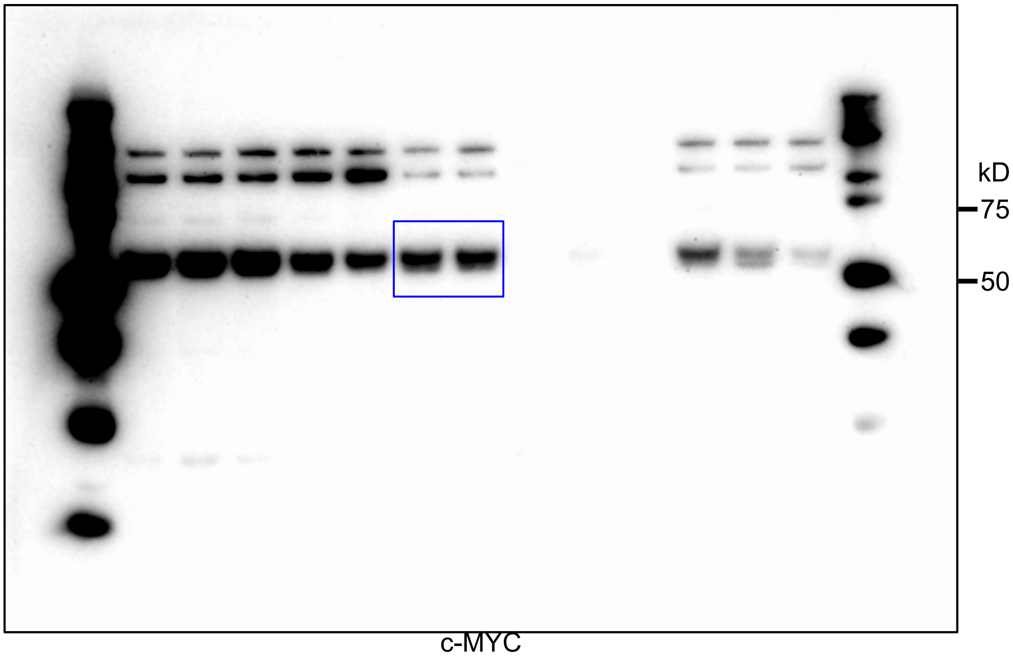

c-MYC

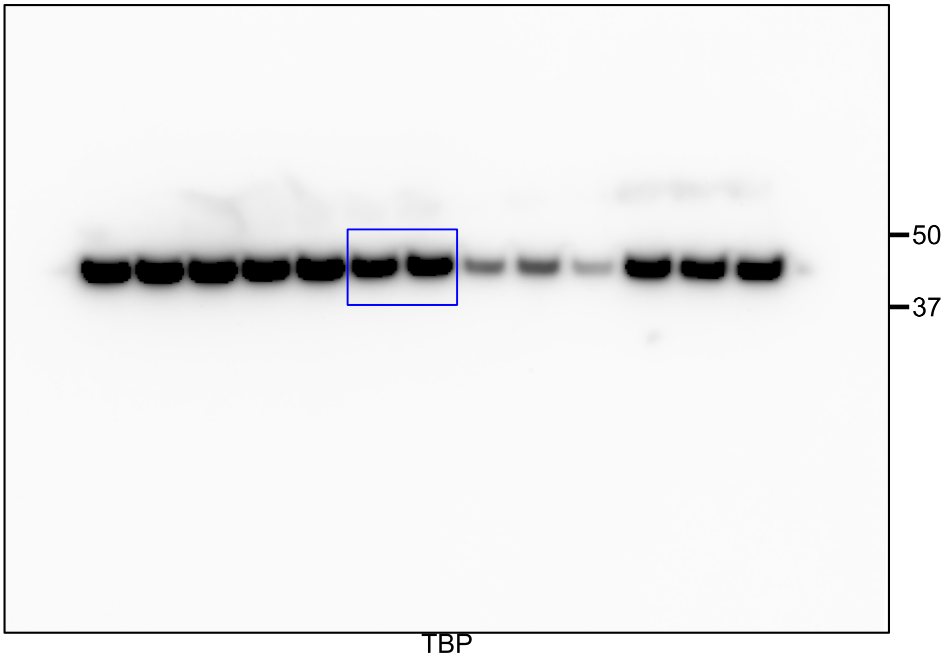

TBP
